# Mutant *C. elegans* mitofusin leads to selective removal of mtDNA heteroplasmic deletions across generations to maintain fitness

**DOI:** 10.1186/s12915-022-01241-2

**Published:** 2022-02-09

**Authors:** Lana Meshnik, Dan Bar-Yaacov, Dana Kasztan, Tali Neiger, Tal Cohen, Mor Kishner, Itay Valenci, Sara Dadon, Christopher J. Klein, Jeffery M. Vance, Yoram Nevo, Stephan Züchner, Ofer Ovadia, Dan Mishmar, Anat Ben-Zvi

**Affiliations:** 1 Department of Life Sciences, Ben-Gurion University of the Negev, Beer Sheva, Israel; 2 Department of Microbiology, Immunology and Genetics, Ben-Gurion University of the Negev, Beer Sheva, Israel; 3 Department of Neurology, Department of Laboratory Medicine and Pathology, Mayo Clinic, Rochester, MN USA; 4 Dr. John T. Macdonald Foundation Department of Human Genetics and Hussman Institute for Human Genomics, Miller School of Medicine, University of Miami, Miami, FL USA; 5 Institute of Neurology, Schneider Children’s Medical Center of Israel, Tel-Aviv University, Petach Tikva, Israel

**Keywords:** *C. elegans*, *fzo-1*, Heteroplasmy inheritance, Mitofusin, mtDNA, PARKIN, *pdr-1*

## Abstract

**Background:**

Mitochondrial DNA (mtDNA) is present at high copy numbers in animal cells, and though characterized by a single haplotype in each individual due to maternal germline inheritance, deleterious mutations and intact mtDNA molecules frequently co-exist (heteroplasmy). A number of factors, such as replicative segregation, mitochondrial bottlenecks, and selection, may modulate the exitance of heteroplasmic mutations. Since such mutations may have pathological consequences, they likely survive and are inherited due to functional complementation via the intracellular mitochondrial network. Here, we hypothesized that compromised mitochondrial fusion would hamper such complementation, thereby affecting heteroplasmy inheritance.

**Results:**

We assessed heteroplasmy levels in three *Caenorhabditis elegans* strains carrying different heteroplasmic mtDNA deletions (ΔmtDNA) in the background of mutant mitofusin (*fzo-1*). Animals displayed severe embryonic lethality and developmental delay. Strikingly, observed phenotypes were relieved during subsequent generations in association with complete loss of ΔmtDNA molecules. Moreover, deletion loss rates were negatively correlated with the size of mtDNA deletions, suggesting that mitochondrial fusion is essential and sensitive to the nature of the heteroplasmic mtDNA mutations. Introducing the ΔmtDNA into a *fzo-1*;*pdr-1;*+/ΔmtDNA (PARKIN ortholog) double mutant resulted in a skewed Mendelian progeny distribution, in contrast to the normal distribution in the *fzo-1;*+/ΔmtDNA mutant, and severely reduced brood size. Notably, the ΔmtDNA was lost across generations in association with improved phenotypes.

**Conclusions:**

Taken together, our findings show that when mitochondrial fusion is compromised, deleterious heteroplasmic mutations cannot evade natural selection while inherited through generations. Moreover, our findings underline the importance of cross-talk between mitochondrial fusion and mitophagy in modulating the inheritance of mtDNA heteroplasmy.

**Supplementary Information:**

The online version contains supplementary material available at 10.1186/s12915-022-01241-2.

## Background

Unlike the nuclear genome, mitochondrial DNA (mtDNA) is present in multiple copies per animal cell. For instance, each human somatic cell contains an average of ~ 1000 mitochondria, with each mitochondrion harboring 1–10 mtDNA copies [1]. Although this intracellular mtDNA population is inherited from the maternal germline and hence carries a single major haplotype, mtDNA molecules can differ in sequence (heteroplasmy) either due to inheritance of mutations from the ovum or due to the accumulation of changes over an individual lifetime [2–5]. Some of these changes may have pathological consequences [6, 7], as reflected in a variety of mitochondrial disorders, yet only upon crossing a threshold of prevalence in the cell [8]. Accordingly, the penetrance of disease-causing mutations ranges between 60 and 80%, depending on the symptoms and tissues that display the specific phenotype [9].

The repertoire of heteroplasmic mutations varies among cells and tissues, mainly due to replicative segregation (drift) of the mitochondria during cell division and mitochondrial bottlenecks that appear during embryo development [10]. However, it has been suggested that heteroplasmy can be modulated by non-random factors, including selection [2, 4, 5, 11, 12]. Indeed, it has been shown that mitophagy, a mechanism of mitochondrial quality control, partially provides selection against defective mitochondria and maintains disease-causing mtDNAs below the threshold levels both in human cells [13] and in a *Caenorhabditis elegans* model system [14–17]. Mitophagy requires proper fission-fusion cycles of the mitochondrial network to allow the removal of dysfunctional mitochondria [18, 19]. In agreement with this notion, elevated heteroplasmy levels of pathological mtDNA molecules were observed when the fission machinery was disrupted in cell culture [20]. Furthermore, reduction in heteroplasmy levels of potentially deleterious mtDNA mutations was observed when components of the fusion machinery were compromised in *Drosophila* model systems, especially in germ cells [12, 21, 22]. In consistence with this notion, cell culture experiments revealed that a mixture of mtDNA molecules differing in sequence in the same cell can complement each other by the diffusion of products via the mitochondrial network, which in turn leads to restoration of mitochondrial function [1, 15, 16, 23]. Hence, mitochondrial fusion likely allows the survival of mtDNA disease-causing mutations in cells and, in turn, their transmission to the next generation [1, 8, 24]. Although these experiments suggest a molecular mechanism for the control of heteroplasmy, it remains unclear whether such a mechanism also affects the transmission of heteroplasmy through generations. Investigating this problem will allow explaining the relatively high abundance of low-level disease-causing heteroplasmic mutations in the general population [25, 26]. We, therefore, hypothesized that interfering with the intracellular mitochondrial network by compromising the fusion machinery would hamper mitochondrial functional complementation and thus impede the inheritance of heteroplasmic mutants.

Here, we took the first steps towards testing this hypothesis by crossing *C. elegans* harboring mitofusin mutant (*fzo-1*) to animals carrying either of three heteroplasmic mtDNA deletions, which differed in size and mtDNA positions. These experiments resulted in embryonic lethality and developmental delay, which were alleviated in subsequent generations concomitant with a complete loss of the truncated mtDNA molecules. Since the rate of truncated mtDNA loss diverged between the heteroplasmic strains, the sensitivity of the fusion machinery to different mtDNA mutations, in addition to its interaction with mitophagy and relevance to human diseases are discussed.

## Results

### A heteroplasmic deletion reduces the fitness of C. elegans mitofusin (fzo-1) mutant

The stable heteroplasmic *C. elegans* strain *uaDf5/+* harbors a mixture of intact (+mtDNA) and ~ 60% of a 3.1 kb mtDNA deletion (ΔmtDNA) [27]. Although lacking four essential genes (i.e., mt-ND1, mt-ATP6, mt-ND2, and mt-Cytb) and seven tRNAs (i.e., K, L, S, R, I, Q and F), this strain is viable and displays some mitochondrial dysfunction [16, 27, 28]. High heteroplasmy levels are likely not maintained due to mtDNA duplication but by stably maintaining +mtDNA copy number [16]. We showed that dysfunctional PDR-1, the worm orthologue of the key mitophagy factor Parkin (PARK2), led to elevated levels of the truncated mtDNA, suggesting that mitochondrial quality control can modulate the levels of dysfunctional mitochondria [14]. In conjunction with this finding, RNAi knockdown of *fzo-1*, the *C. elegans* orthologue of MFN1/2, led to a slight reduction in the levels of the heteroplasmic ΔmtDNA, although without any phenotypic consequences [15]. We, therefore, asked what would be the impact of the *fzo-1(tm1133)* deletion (hereafter designated as *fzo-1(mut)*) on the inheritance of the ΔmtDNA.

**Fig. 1:**
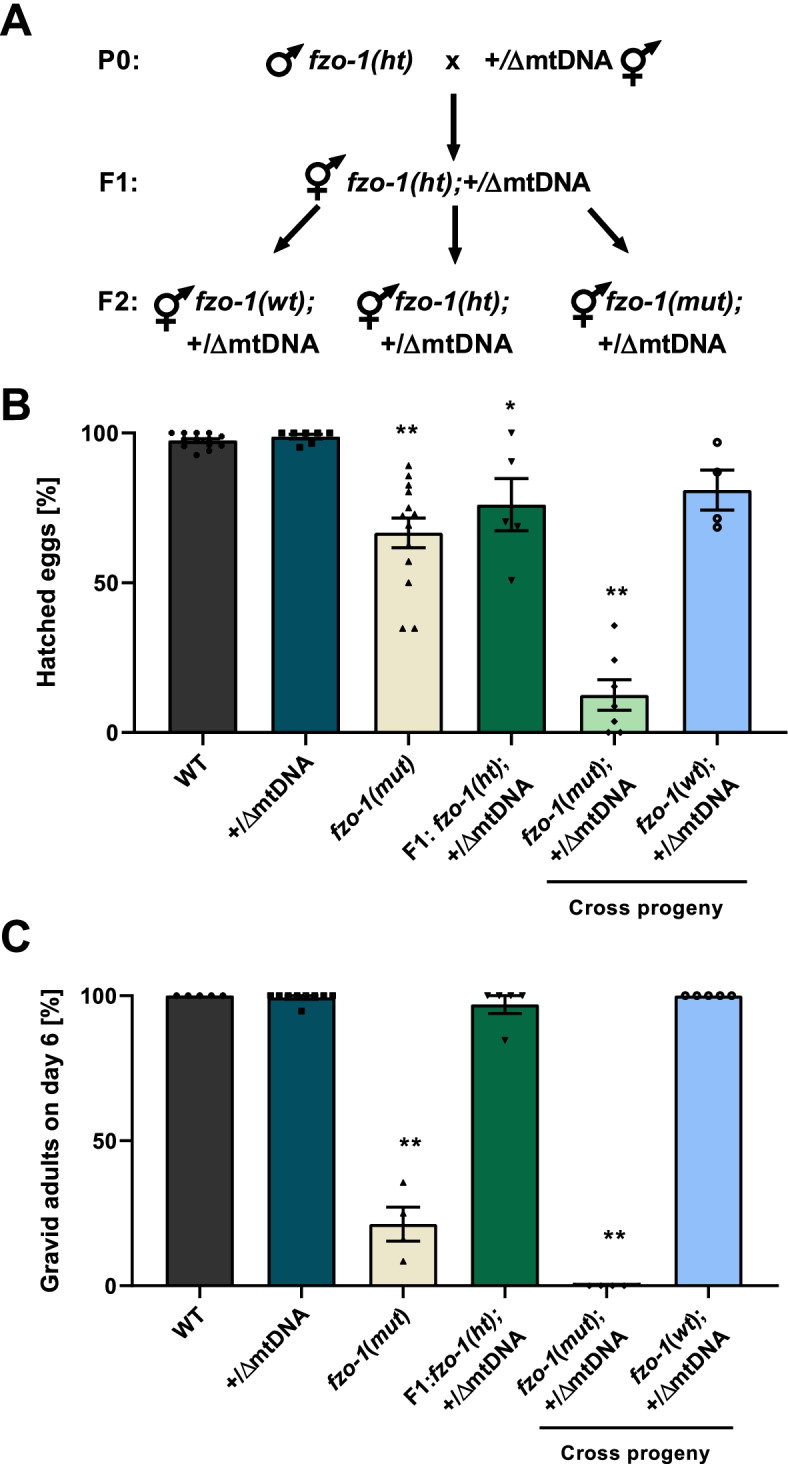
ΔmtDNA reduces the fitness of *fzo-1(mut)* animals. **A** Establishing *fzo-1* mutant and wild type heteroplasmic lines. Heteroplasmic hermaphrodites carrying intact and truncated mtDNA [*uaDf5/+*] were crossed with *fzo-1(tm1133)* heterozygotes males, *fzo-1(ht)*. Cross progeny F1 were allowed to self-propagate. Single F2 animals were isolated, allowed to lay eggs, and their genotypes were determined using a single worm PCR. Heteroplasmic (+/ΔmtDNA) mutant *(mut)* or wild type (*wt*) *fzo-1* progeny were then monitored. **B** The percent of hatched embryos of parental strains: N2 (WT; *N* = 13, *n* = 869), +/ΔmtDNA (*N* = 7, *n* = 265) and *fzo-1(mut)* (*N* = 13, *n* = 386), of *fzo-1(ht);*+/ΔmtDNA animals (*N* = 5, *n* = 152) and of mutant, *fzo-1(mut);*+/ΔmtDNA (*N* = 7, *n* = 236) or wild type *fzo-1(wt);*+/ΔmtDNA (*N* = 4, *n* = 104) cross progeny. Data are means ± 1 standard error of the mean (1SE). Data were analyzed using one-way ANOVA followed by a Tukey’s post hoc test. (*) denotes *P* < 0.05 and (**) denotes *P* < 0.001 compared with WT animals. **C** The percent of gravid adults six days after egg laying of parental strains: N2 (WT) (*N* = 5, *n* = 114), +/ΔmtDNA (*N* = 9, *n* = 232) and *fzo-1(mut)* (*N* = 4, *n* = 278), of *fzo-1(ht);*+/ΔmtDNA animals (*N* = 5, *n* = 69), and of mutant, *fzo-1(mut);*+/ΔmtDNA (*N* = 4, *n* = 152) or wild type *fzo-1(wt);*+/ΔmtDNA (*N* = 5, *n* = 245) cross progeny. Data are means ±1 standard error of the mean (1SE). Data were analyzed using one-way ANOVA followed by a Tukey’s post hoc test. (**) denotes *P* < 0.001 compared with WT animals. Individual data values are presented in Additional file 2

To this end, we crossed *uaDf5/+* heteroplasmic hermaphrodites (+/ΔmtDNA) with *fzo-1(mut)* heterozygote males (Fig. 1A). After self-cross of the F1 progeny, the distribution of the genotypes in the F2 heteroplasmic progeny did not deviate from the expected Mendelian ratio, namely 26% homozygous *fzo-1(mut)*, 48.7% *fzo-1* heterozygotes (*ht*), and 25.3% *fzo-1* wild type *(wt)* (chi-square test, *P* = 0.960; Additional file 1: Table S1). However, we noticed that only 13 ± 5% of the progeny of the self-crossed *fzo-1(mut);*+/ΔmtDNA worms hatched, as compared to *fzo-1(mut)* animals (67 ± 5%, ANOVA followed by a Tukey’s post hoc test, *P* < 0.001; Fig. 1B). Although mitochondrial organization and TMRE uptake of self-crossed *fzo-1(mut);*+/ΔmtDNA adults were similar to *fzo-1(mut)* (Additional file 1: Fig. S1A-B), *fzo-1(mut);*+/ΔmtDNA animals were developmentally delayed, and none of them reached adulthood after six days as compared to *fzo-1(mut)* (ANOVA followed by a Tukey’s post hoc test, *P* < 0.001). This was in contrast to self-crossed *fzo-1(wt);*+/ΔmtDNA animals, all of which reached adulthood after six days (Fig. 1C). These findings demonstrate that the interaction between the heteroplasmic ΔmtDNA and the nuclear DNA-encoded *fzo-1* mutant led to a severe reduction in fitness.

### The adverse effects of the interaction between ΔmtDNA and fzo-1(mut) are reversed across generations

**Fig. 2:**
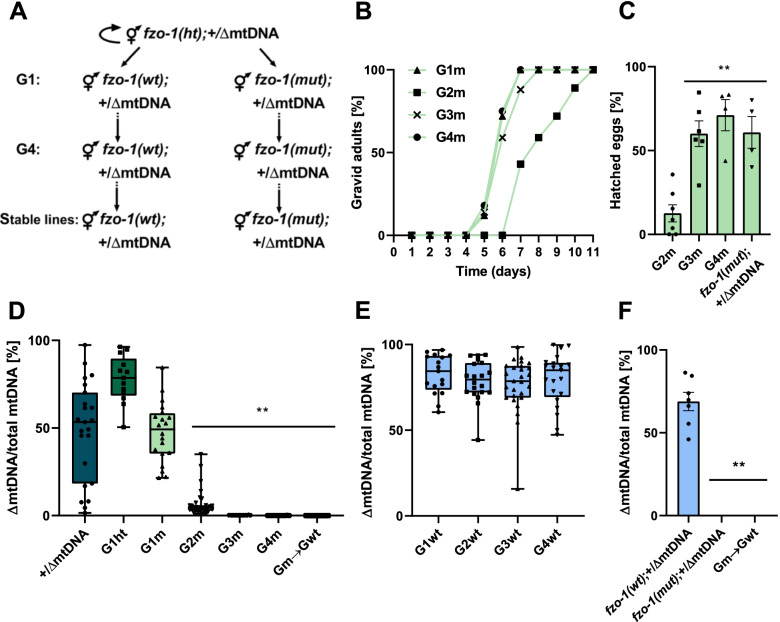
ΔmtDNA levels are selectively eliminated, and their adverse effects reversed in *fzo-1(mut)*;+/ΔmtDNA animals across generations. **A** Schematic representation of the experimental setup. The *fzo-1* heterozygotes progeny of heteroplasmic hermaphrodites (*fzo-1(ht)*;+/ΔmtDNA*)* was identified and maintained using self-propagation and single worm genotyping to establish heteroplasmic lines carrying *fzo-1(ht);*+/ΔmtDNA. Progeny animals (generation 1; G1) were isolated, allowed to lay eggs, and their genotypes were determined. Heteroplasmic mutant *fzo-1(mut)*;+/ΔmtDNA or wild type *fzo-1(wt);*+/ΔmtDNA progeny were then monitored over several generations (G2m-G4m and G2wt-G4wt, respectively). **B** The percent of gravid adults of *fzo-1(mut)*;+/ΔmtDNA mutant progeny across generations (G1m-G4m) at the indicated times after egg laying (G1m *N* = 3, *n* = 40, G2m *N* = 4, *n* = 152, G3m *N* = 3, *n* = 155 and G4m *N* = 3, *n* = 82). Data were analyzed using Cox proportional-hazards regression (Additional file 1: Table S2). G2m and G3m were slower to reach adulthood than G1m (*P* < 0.001) but not G4m (*P* = 0.750). **C** The percent of hatched embryos of *fzo-1(mut);*+/ΔmtDNA progeny across generations (G2m *N* = 7, *n* = 236, G3m *N* = 6, *n* = 155 and G4m *N* = 4, *n* = 107) and the stable line (> 20 generations) *fzo-1(mut);*+/ΔmtDNA (*N* = 4, *n* = 93). Data are means ±1 standard error of the mean (1SE). Data were analyzed using one-way ANOVA followed by a Tukey’s post hoc test, (**) denotes *P* < 0.002 compared with G2m animals. **D**, **E** Box plot showing the percent of ΔmtDNA (*N* > 3 biological repeats) determined in individual animals (**D**) of the parental heteroplasmic strain +/ΔmtDNA (*n* = 23), the heteroplasmic *fzo-1(mut)* mutant cross-progeny strains (G1ht *n* = 13, G1m-G4m *n* = 20, 31, 21 and 21, respectively) and the progeny of G4m animals crossed with *fzo-1(wt)*, (Gm→Gwt *n* = 21); (**E**) of the heteroplasmic *fzo-1(wt)* cross progeny strains (G1wt-G4wt *n* = 17, 20, 27 and 21, respectively). In the boxplot representation, center line, median; box limits, upper and lower quartiles; whiskers, minimum and maximum; points, data. Data were analyzed using Fractional regression (Additional file 1: Table S3). ΔmtDNA levels of G2m-G4m and Gm->Gwt were significantly lower than those of the parental heteroplasmic strain +/ΔmtDNA, (**) denotes *P* < 0.001. **F** The percent of ΔmtDNA determined for a population of animals from the stable cross lines (> 20 generations), *fzo-1(wt);*+/ΔmtDNA (*N* = 7), *fzo-1(mut)*;+/ΔmtDNA (*N* = 3) and +/ΔmtDNA;*fzo-1(Gm*→*Gwt)* (*N* = 4). Data are means ±1 standard error of the mean (1SE). Data were analyzed using one-way ANOVA followed by a Tukey’s post hoc test, (**) denotes *P* < 0.001 compared with *fzo-1(wt);+/*ΔmtDNA animals. Individual data values are presented in Additional file 2

To better characterize the phenotypic impact of the interactions between ΔmtDNA and *fzo-1(mut)*, we monitored the development of progeny of the self-crossed *fzo-1(ht);*+/ΔmtDNA worms, followed by genotyping the resultant adult animals (generation 1, G1). We continued to follow the hatching and development of their progeny, i.e., *fzo-1(mut);*+/ΔmtDNA (G2m) and *fzo-1(wt);*+/ΔmtDNA (G2wt), across four generations (Fig. 2A). Specifically, we measured the duration of the larva-to-adulthood period during development in the G1m-G4m generations (Fig. 2B). While ~ 75% of the G1m animals reached adulthood after 6 days, the development of G2m animals was 1.9-fold delayed (Cox proportional-hazards regression, *P* < 0.001), with 75% of the animals reaching adulthood only after 9 days. Surprisingly, the G3m animals showed significant improvement (Cox proportional-hazards regression, *P* < 0.001), with ~ 60% of this population reaching adulthood after six days. Moreover, G4m animals showed a full reversal of ΔmtDNA-associated adverse effects (Cox proportional-hazards regression, *P* = 0.750; Fig. 2B and Additional file 1: Table S2). We noted a similar pattern across generations when hatching was considered: In contrast to the 13.5% hatching observed among G2m embryos, 60 ± 8% hatching of the G3m embryos was observed (ANOVA followed by a Tukey’s post hoc test, *P* < 0.001). The hatching percentage of the G4m generation was similar to that of G1 animals (71 ± 9% and 67 ± 5%, respectively, ANOVA followed by a Tukey’s post hoc test *P* = 0.993) and remained stable over subsequent generations (Fig. 2C). Finally, no phenotypic changes were observed for G1wt-G4wt animals while tracing their developmental pace (Cox proportional-hazards regression, *P* > 0.140; Additional file 1: Table S2) and hatching percentage (ANOVA followed by a Tukey’s post hoc test, *P* > 0.266; Additional file 1: Fig. S2A-B). Taken together, our findings demonstrate a full reversal of the adverse effects of the interaction between the ΔmtDNA and the nuclear DNA-encoded mutant *fzo-1* gene.

### fzo-1(mut) leads to selection against ΔmtDNA heteroplasmy in C. elegans

We next asked how the deleterious interactions between *fzo-1(mut)* and ΔmtDNA were abrogated. We hypothesized that if the ΔmtDNA is not tolerated in the background of *fzo-1(mut)*, then selection against the ΔmtDNA should occur. To test this prediction, we assessed the levels of ΔmtDNA by quantitative PCR (qPCR, see Methods) across the G1m-G4m generations (using gravid adults) in both the *fzo-1(mut);*+/ΔmtDNA and the *fzo-1(wt);*+/ΔmtDNA strains. We found that ΔmtDNA levels declined by 10-fold in the G2m *fzo-1(mut)* animals, as compared to +/ΔmtDNA parental strain (Fractional regression, odds ratio = 0.08; *P* < 0.001). Values of ΔmtDNA reached below detection levels in most G3m (*N* = 18/21) and G4m (*N* = 20/21) animals (fractional regression, odds ratio < 0.007, *P* < 0.001; Fig. 2D and Additional file 1: Table S3), while the relative levels of intact +mtDNA molecules increased (Additional file 1: Fig. S2C and Table S3). In contrast, ΔmtDNA and +mtDNA levels did not significantly change across the G1wt-G4wt generations of *fzo-1(wt)*;+*/*ΔmtDNA animals (fractional regression, *P* = 0.852; Fig. 2E and Additional file 1: S2D and Table S3).

These results suggest that the ΔmtDNA was completely lost during the G1m-G4m generations. To test this hypothesis, we crossed G4m hermaphrodites with wild type males to isolate *fzo-1(wt)* progeny (Gm→Gwt). Since traces of ΔmtDNA were neither detected in Gm->Gwt animals (fractional regression, *P* < 0.001; Fig. 2D) nor in subsequent generations (ANOVA followed by a Tukey’s post hoc test, *P* < 0.001; Fig. 2F), we concluded that disrupting *fzo-1* function indeed resulted in a complete and specific loss of the deleterious heteroplasmic ΔmtDNA. These results provide a proof of concept that mitochondrial fusion is critical for regulating the transmission of the *uaDf5* ΔmtDNA heteroplasmy across generations.

### Selection against heteroplasmic truncations depends on deletion size or mtDNA position

We next asked whether the deleterious interactions between *fzo-1(mut)* and ΔmtDNA depend on the size or genomic location of mtDNA deletions. To achieve this goal, we characterized two additional mtDNA deletions obtained from the Million Mutation Project strain collection [29]. Specifically, these deletions comprise two new stable heteroplasmic *C. elegans* strains: *bguDf1* (derived from strain VC40128), harboring a mixture of intact +mtDNA along with mtDNA molecules lacking ~ 1 kb (1kbΔmtDNA) encompassing two essential mtDNA genes (i.e., mt-ATP6 and mt-ND2) and three tRNAs (i.e., K, L, and S); the second strain, *bguDf2* (derived from VC20469), harbors in addition to the +mtDNA, a ~4.2 kb mtDNA deletion (4kbΔmtDNA) encompassing four different essential genes (i.e., mt-CO1, mt-CO2, mt-ND3, and mt-ND5) and five tRNAs (i.e., C, M, D, G, and H). Notably, the levels of the 1kbΔmtDNA and 4kbΔmtDNA were stable over > 100 generations (80% and 55%, respectively) in the presence of functional (wild type) *fzo-1*. Reanalysis of whole-genome sequencing data for the two mutant strains identified +mtDNA and deletion sequences, as previously described [29, 30]. These analyses did not reveal any evidence for duplicated regions, confirming the mtDNA deletion heteroplasmy in these strains (Additional file 1: Fig. S3A-B). As previously observed for *uaDf5/+* [16], truncated mtDNA levels highly varied between animals, while intact +mtDNA levels were more constant (Additional file 1: Fig. S3C-D). The animals displayed neither embryonic nor developmental phenotypes, and no impact on mitochondria fusion was observed (Additional file 1: Fig. S3E-G and Table S2).

Heteroplasmic hermaphrodites of both strains were separately crossed with *fzo-1(mut)* heterozygote males, followed by self-cross of F1 progeny (cross as in Fig. 1A). Like the approach taken with the *uaDf5/+* strain*,* we examined the distribution of the genotypes in the F2 heteroplasmic progeny. We found that the genotypes distribution for the *fzo-1(mut);*+/1kbΔmtDNA did not deviate from the expected Mendelian ratio (23.5% homozygous *fzo-1(mut),* 43.2% *fzo-1*(*ht*) and 33.3% *fzo-1(wt)* (*P* = 0.14, chi-square test; Additional file 1: Table S1). In contrast, this ratio strongly deviated from the expected Mendelian ratio for *fzo-1(mut)* animals harboring +/4kbΔmtDNA (6.4% homozygous *fzo-1(mut)*, 59.3% *fzo-1*(*ht*) and 34.3% *fzo-1(wt)* (*P* < 0.001, chi-square test; Additional file 1: Table S1). Hence, these results indicate that *fzo-1(mut)* differentially tolerates mtDNA deletions based on size and/or mtDNA position*.*

**Fig. 3:**
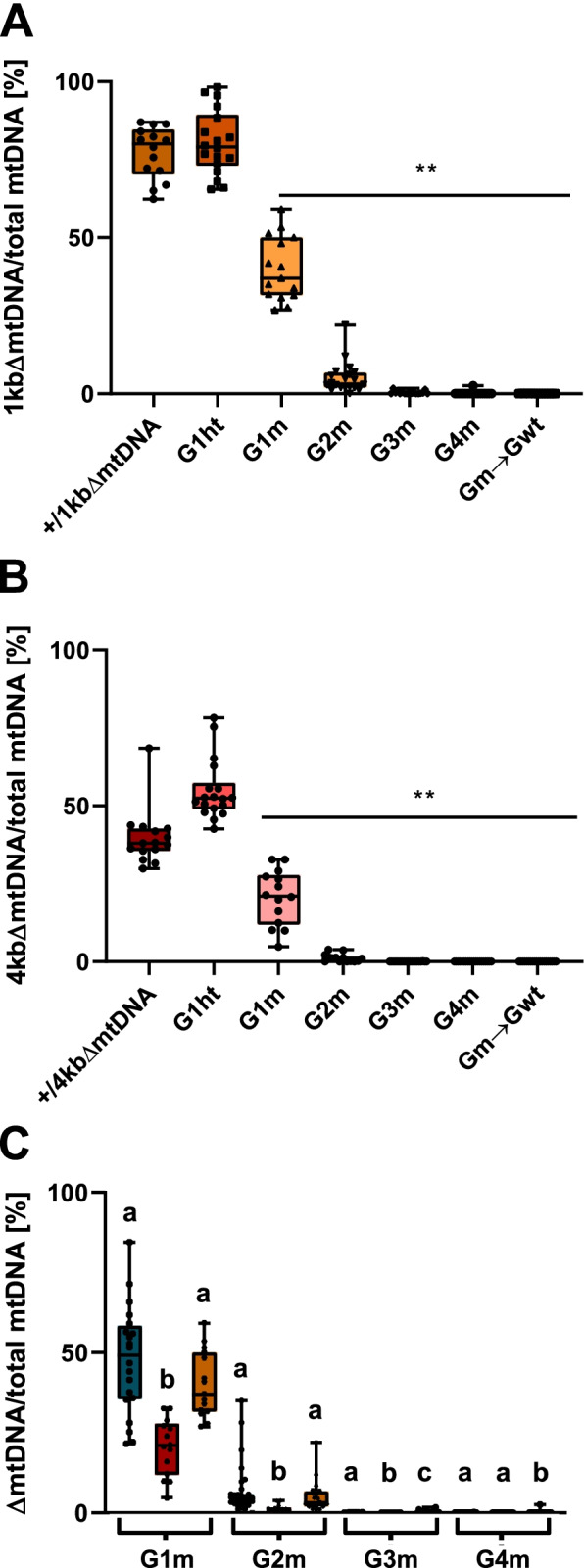
ΔmtDNA levels are differentially eliminated in *fzo-1(mut)*;ΔmtDNA animals based on size or mtDNA position. **A**, **B** Box plot showing the percentage of 1kbΔmtDNA (**A**) or 4kbΔmtDNA (**B**) ΔmtDNA (*N* > 3 biological repeats), determined in individual animals of the parental heteroplasmic strain +/1kbΔmtDNA (*n* = 14) and +/4kbΔmtDNA (*n* = 15), the *fzo-1(mut)* mutant cross-progeny strains (1kbΔmtDNA F1(ht) *n* = 18, G1m-G4m *n* = 15, 20, 17 and 19 and 4kbΔmtDNA F1(ht) *n* = 18, G1m-G4m *n* = 14, 14, 21 and 20) and the progeny of G4m animals crossed with *fzo-1(wt)*, (Gm → Gwt *n* = 15 and *n* = 20 respectively). In the boxplot representation, center line, median; box limits, upper and lower quartiles; whiskers, minimum and maximum; points, data. Data were analyzed using fractional regression (Additional file 1: Table S3). ΔmtDNA levels of G1m-G4m and Gm->Gwt were significantly lower than those of the parental strains in both +/1kbΔmtDNA and +/4kbΔmtDNA, (**) denotes *P* < 0.001. **C** Box plot comparing the percentage of ΔmtDNA in animals carrying +/3kbΔmtDNA (blue), +/1kbΔmtDNA (yellow) or +/4kbΔmtDNA (red) in each generation (G1m-G4m; data from Fig. 2C, Fig. 3A, and Fig. 3B, respectively). In the boxplot representation, center line, median; box limits, upper and lower quartiles; whiskers, minimum and maximum; points, data. Data were analyzed using fractional regression followed by within generation pairwise comparisons (Additional file 1: Table S3), different letters indicate a significant difference in mean levels of ΔmtDNA levels: G1-G2, 4 kb levels (a) lower than 3 kb and 1 kb (b, *P* < 0.001); G3, 4 kb levels (a) lower than 3 kb (b, *P* < 0.01) and 4 kb (a) and 3 kb (b) levels lower than 1 kb (c, *P* < 0.001); G4, 4 kb and 3 kb levels (a) lower than 1 kb (b, *P* < 0.001). Individual data values are presented in Additional file 2

We next quantified the levels of ΔmtDNA in mutant versus wild type *fzo-1* progeny across four subsequent generations (as in Fig. 2A; Fig. 3 and Additional file 1: Table S3). We found that both truncated mtDNA molecules were undetectable after four generations (Fig. 3A, B), yet the decline rates significantly diverged (Fig. 3C). Specifically, mean ΔmtDNA levels were significantly lower in *fzo-1(mut)* animals harboring the 4kbΔmtDNA than in animals harboring either 1kbΔmtDNA or 3kbΔmtDNA in both G1m and G2m animals (fractional regression followed by within generation pairwise comparisons, *P* < 0.001; Fig. 3C and Additional file 1: Table S3). By the G3m generation, mean ΔmtDNA levels of 3kbΔmtDNA were also significantly lower than those observed in animals harboring 1kbΔmtDNA. Indeed, the 1kbΔmtDNA was still detected in most of G3m animals (*N* = 13/18). It is worth noting that the levels of both types of ΔmtDNA did not significantly change across the G1wt-G4wt generations of the *fzo-1(wt);*+/ΔmtDNA animals (fractional regression, *P* > 0.118 in both cases; Additional file 1: Fig. S3H-I and Table S3).

To assess whether the truncated mtDNAs were completely lost, we crossed G4m hermaphrodites with wild type males and isolated *fzo-1(wt)* progeny (Gm→wt). qPCR analyses revealed no traces of the ΔmtDNA copies in subsequent generations (fractional regression, *P* < 0.001; Fig. 3A-B and Additional file 1: Table S3). Thus, disrupting *fzo-1* function resulted in a complete and specific loss of a variety of heteroplasmic ΔmtDNAs. We interpret these results to mean that *fzo-1* function is sensitive to either the size or location of deleterious mtDNA heteroplasmy.

### Selection against ΔmtDNA molecules occurs during C. elegans development

**Fig. 4:**
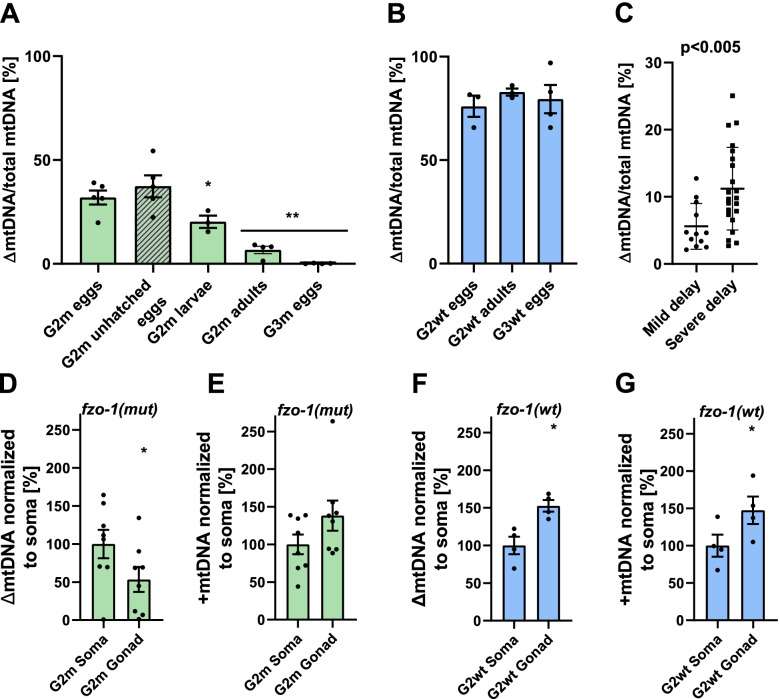
ΔmtDNA molecules are selectively eliminated during *C. elegans* development and defer between gonad and soma. **A** The percent of ΔmtDNA determined for a population of animals in generation 2 mutant, G2m, eggs (*N* = 5), unhatched eggs (*N* = 5), larvae (*N* = 3) and adults (*N* = 4) and generation 3 mutant, G3m eggs (*N* = 4). Data are means ±1 standard error of the mean (1SE). Data were analyzed using one-way ANOVA followed by a Tukey’s post hoc test, (*) denotes *P* < 0.05 and (**) denotes *P* < 0.001 compared with G2m unhatched eggs. **B** The percent of ΔmtDNA determined for a population of animals (*n* = 5) in generation 2 wild type, G2wt eggs (*N* = 3) and adults (*N* = 3), and generation 3 wild type, G3wt eggs (*N* = 4). Data are means ± 1 standard error of the mean (1SE). Data were analyzed using one-way ANOVA followed by a Tukey’s post hoc test compared with G2wt eggs. **C** The percent of ΔmtDNA determined in individual G2m adults that reached adulthood (*N* = 3 biological repeats) after 7–8 days (mild delay; *n* = 12) or 9–10 days (severe delay; *n* = 22). Data were analyzed using the Wilcoxon Mann-Whitney rank sum test (*P* < 0.005). **D**–**G** The relative levels of ΔmtDNA (**D**, **F**) or +mtDNA (**E**, **G**) were determined for the gonad and soma (normalized to soma) of a population of (**D**, **E**) G2m adults (*N* = 8) or (**F**, **G**) G2wt adults (*N* = 4). Data were analyzed using the Wilcoxon Mann-Whitney rank sum test, (*) denotes *P* < 0.05. Individual data values are presented in Additional file 2

In *C. elegans,* mtDNA copy numbers increase significantly during the fourth larval stage (L4) in association with oocyte production [27, 31]. We, therefore, asked at which point during the *C. elegans* life cycle selection against ΔmtDNA occurred. Given that the relative levels of ΔmtDNA are maintained during normal development [27], we compared ΔmtDNA levels between embryos and adults in G2m animals. Our results indicate that ΔmtDNA levels were dramatically reduced (~ 5-fold) during the development of G2m animals (ANOVA followed by a Tukey’s post hoc test, *P* < 0.001; Fig. 4A) but not in G2wt animals (Fig. 4B). This observation suggests that ΔmtDNA is most likely selected against during the *fzo-1(mut)* worm development, in agreement with the observed adverse effect of heteroplasmy on the hatching and development of G2m animals.

To examine the possible association of ΔmtDNA levels with embryo lethality, we compared ΔmtDNA levels of unhatched embryos (unhatched > 48 h after being laid) to newly hatched larvae (L1). As expected, given that ~ 85% of G2 embryos did not hatch, ΔmtDNA levels of unhatched embryos were similar to the relative ΔmtDNA levels of newly laid embryos. In contrast, ΔmtDNA levels in L1 animals were reduced by 2-fold (ANOVA followed by a Tukey’s post hoc test, *P* < 0.05; Fig. 4A). This suggests that hatching is enabled only in embryos with reduced ΔmtDNA levels.

We next examined whether ΔmtDNA levels associate with developmental delay. To this end, we compared the levels of ΔmtDNA in mildly delayed G2 animals that reached adulthood on days 7–8 to severely delayed animals that reached adulthood on days 9–10. Our results show that ΔmtDNA levels were 2-fold higher in the severely delayed group (Wilcoxon Mann-Whitney rank sum test, *P* < 0.005 test; Fig. 4C). These data demonstrate increased embryo lethality and aggravation in developmental delay in animals harboring high levels of ΔmtDNA. This supports our interpretation that disruption of mitochondrial fusion in animals carrying ΔmtDNA molecules leads to reduced fitness and suggests that there is selection against such molecules at the level of the organism.

### Selection against ΔmtDNA molecules defers between gonad and soma

Previously Lieber et al. demonstrated germline selection acting against high levels of mutant mtDNA in *Drosophila* oogenesis [22]. In *C. elegans,* the germline tends to accumulate higher levels of deleterious mitochondrial molecules than somatic tissues, although unfertilized oocytes contain lower levels of ΔmtDNA compared to that of germline tissue [32]. Consistently, we found that ΔmtDNA molecules became undetectable in the resultant embryos of G2m animals (i.e., in G3m animals; Fig. 4A). Hence, it is possible that selection against ΔmtDNA molecules occurred during *C. elegans* gametogenesis. In support of this claim, a comparison of ΔmtDNA levels between gonads and somatic tissues in G2m animals revealed a two-fold decrease of ΔmtDNA levels in the gonads (Wilcoxon Mann-Whitney rank sum test, *P* < 0.05; Fig. 4D), whereas the levels of +mtDNA intact molecules were ~1.4-fold higher in the gonad (Fig. 4E). In contrast, both ΔmtDNA and +mtDNA molecules were ~1.5-fold higher in the gonad when comparing gonads and somatic tissues of *fzo-1(wt)*;+/ΔmtDNA animals (*P* < 0.05 Wilcoxon Mann-Whitney rank sum test; Fig. 4F, G) [32]. Since both the *Drosophila* experiments [12, 21, 22] and our observations are consistent, we argue that selection against ΔmtDNA molecules during gametogenesis is evolutionarily conserved [12].

### PARKIN mutant aggravates the adverse effects of the ΔmtDNA-fzo-1 interactions

**Fig. 5:**
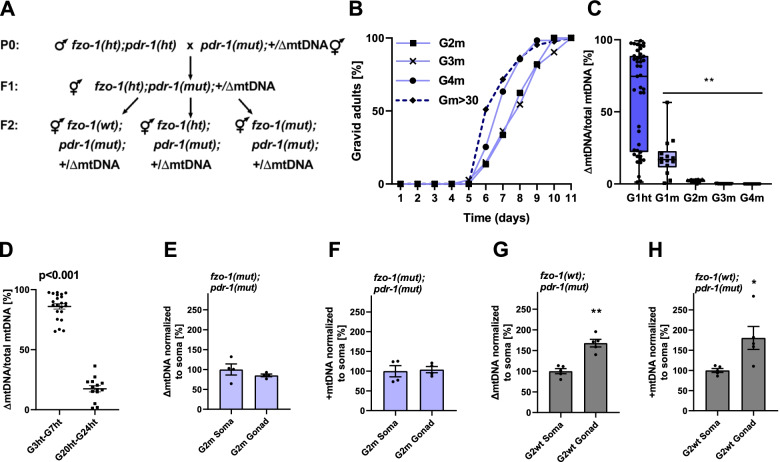
Mutant Parkin aggravates the adverse effects of the ΔmtDNA;*fzo-1*. **A** Schematic representation of experimental setup. The *fzo-1* heterozygotes progeny of heteroplasmic *pdr-1(mut*) hermaphrodites (*fzo-1(ht);pdr-1(mut);+/*ΔmtDNA) was identified and maintained using self-propagation and single worm genotyping to establish heteroplasmic lines carrying *fzo-1(ht);pdr-1(mut);+/*ΔmtDNA. Progeny animals (generation 1; G1) were isolated, allowed to lay eggs and their genotypes were determined. The progeny of heteroplasmic *pdr-1(mut*) hermaphrodites that were *fzo-1* mutant (*fzo-1(mut);pdr-1(mut);*+/ΔmtDNA) or wild type (*fzo-1(wt);pdr-1(mut);+/*ΔmtDNA) were then monitored over several generations (G2m-G4m and G2wt-G4wt, respectively). **B** The percent of gravid adults of *fzo-1(mut);pdr-1(mut)*;+/ΔmtDNA mutant progeny across generations (G2m-G4m) and of the stable cross line (> 30 generations, Gm > 30) was determined at the indicated times after egg laying (G2m *N* = 6, *n* = 140, G3m *N* = 5, *n* = 83, G4m *N* = 6 *n* = 89, and Gm > 30 *N* = 3, *n* = 116). Data were analyzed using Cox proportional-hazards regression (Additional file 1: Table S2). G2m-G3m were slower to reach adulthood than Gm > 30 (*P* < 0.001) but not G4m (*P* = 0.084). **C** Box plot showing the percent of ΔmtDNA (*N* > 3 biological repeats) determined in individual animals of the heteroplasmic mutant cross-progeny strains, *fzo-1(mut);pdr-1(mut);*+/ΔmtDNA (G1(ht) *n* = 41, G1m-G4m *n* = 14, 20, 17 and 16, respectively). In the boxplot representation, center line, median; box limits, upper and lower quartiles; whiskers, minimum and maximum; points, data. Data were analyzed using Fractional regression (Additional file 1: Table S3). ΔmtDNA levels of G1m-G4m were significantly lower than those observed in G1(ht), (**) denotes *P* < 0.001. **D** Heteroplasmy levels of individual *fzo-1(ht);pdr-1(mut);+/*ΔmtDNA animals (*N* > 3 biological repeats) sampled at generations 3–7 (Ght3-Ght7; *n* = 22) and generations 20–24 (Ght20-Ght24; *n* = 14). Data were analyzed using the Wilcoxon Mann-Whitney rank sum test (*P* < 0.001). **E**–**H** The relative levels of ΔmtDNA (**E**, **G**) or +mtDNA (**F**, **H**) determined for the gonad and soma (normalized to soma) of a population of **E**–**F** G2m adults (*N* = 4) or **G**–**H** G2wt adults (*N* = 5). Data were analyzed using the Wilcoxon Mann-Whitney rank sum test, (*) denotes *P* < 0.05 and (**) denotes *P* < 0.001. Individual data values are presented in Additional file 2

Since Parkin mediates the turnover of mitofusins and hence impacts their activity [33, 34], we asked what would be the impact of the *pdr-1;fzo1* double mutant on the inheritance of the ΔmtDNA. To address this question, we first crossed +/ΔmtDNA heteroplasmic hermaphrodites with *pdr-1(gk448)* (here named *pdr-1(mut)*) males and established a stable *pdr-1(mut);*+/ΔmtDNA strain. Consistent with previous findings, the ΔmtDNA levels were elevated in this strain (75%) [14–17]. To establish a strain which is mutant in *pdr-1* and *fzo-1* in the context of +/ΔmtDNA heteroplasmy, we then crossed *pdr-1(mut);*+/ΔmtDNA heteroplasmic hermaphrodites with *pdr-1;fzo-1* heterozygous males, let the F1 progeny self-cross, and isolated *fzo-1(ht);pdr-1(mut)* hermaphrodites that are harboring +/ΔmtDNA (Fig. 5A). This strain was allowed to propagate, and the genotypic distribution of the heteroplasmic progeny was assessed. While the genotype distribution of *fzo-1(mut);+/*ΔmtDNA did not deviate from the expected Mendelian ratio, the genotype distribution of *fzo-1(ht);pdr-1(mut);+/*ΔmtDNA was strongly affected, as follows: 9% homozygous *fzo-1(mut);pdr-1(mut)*, 55.8% *fzo-1(ht);pdr-1(mut)*, and 35.2% *fzo-1(wt);pdr-1(mut)* (*P* < 0.001, chi-square test; Additional file 1: Table S1). This suggests that the heteroplasmic ΔmtDNA cannot be tolerated in the background of *fzo-1(mut);pdr-1(mut)* double mutant, as reflected in further reduction in fitness.

We next monitored the development and fecundity of these animals. Phenotypic characterization revealed a developmental delay in the *fzo-1(mut);pdr-1(mut);*+/ΔmtDNA G1m, similar to the parental strain (11/11 were adults by day 7); and most of the G1m progeny (G2m eggs) hatched (85 ± 8%; Additional file 1: Fig. S4A). However, 66 ± 1% of the G2m were developmentally arrested (Additional file 1: Fig. S4B). Only 33 ± 12% of the remaining animals reached adulthood by day 7 (Cox proportional-hazards regression, *P* < 0.001; Fig. 5B and Additional file 1: Fig. S4C and Table S2), and their progeny production was severely reduced (laying seven eggs or less over 20 h). Moreover, 22 ± 7.5% of the G3m animals were still developmentally arrested (Additional file 1: Fig. S4B), and G3m development was similarly delayed (Cox proportional-hazards regression, *P* < 0.001; Fig. 5B and Additional file 1: Fig. S4C and Table S2). However, we noticed a significant recovery of animals’ development during subsequent generations (Fig. 5B and S4C). In contrast, heteroplasmic *fzo-1(wt);pdr-1(mut);*+/ΔmtDNA hatching and development was unaffected (~ 98% hatched and 100% were adults by day 7; Additional file 1: Fig. S4A and S4C). Thus, the adverse effects of the interaction between *fzo-1(mut)* and ΔmtDNA on fecundity and developmental timing were aggravated by *pdr-1(mut)*, supporting *fzo-1-pdr-1* epistasis*.*

We next asked whether the selection against ΔmtDNA would strengthen in the background of *fzo-1(mut);pdr-1(mut)*;+/ΔmtDNA. To directly examine this, we compared the levels of ΔmtDNA molecules in *fzo-1(mut);pdr-1(mut)*;+/ΔmtDNA animals (Fig. 5C) to *fzo-1(mut)*;+/ΔmtDNA (Fig. 2D) across G1m-G4m generations. We found that ΔmtDNA levels declined more sharply in *fzo-1(mut);pdr-1(mut)*;+/ΔmtDNA as compared to the single mutant strain *fzo-1(mut)*;+/ΔmtDNA. Specifically, we found lower ΔmtDNA levels in G1m and G2m *fzo-1(mut);pdr-1(mut)*;+/ΔmtDNA animals (fractional regression followed by within generation pairwise comparisons, *P* < 0.001; Additional file 1: Table S3). In contrast, ΔmtDNA levels did not significantly change across generations of *fzo-1(wt);pdr-1(mut);+/*ΔmtDNA animals (fractional regression, *P* > 0.167 in all cases; Additional file 1: Fig. S4D and Table S3). Taken together, the concomitant disruption of fusion and mitophagy strongly selected against ΔmtDNA molecules. In support of this interpretation, we noticed that even in the *fzo-1(ht);pdr-1(mut)*;+/ΔmtDNA animals, where only one genomic copy of *fzo-1* remained functional, ΔmtDNA levels declined and in some individuals were lost across ~ 25 generations (Wilcoxon Mann-Whitney rank sum test, *P* < 0.001; Fig. 5D).

Finally, we asked whether impaired mitophagy affects selection against ΔmtDNA in the worm germline. To this end, we examined the ratio of ΔmtDNA and +mtDNA between the gonad and soma in double mutant *fzo-1(mut);pdr-1(mut);*+/ΔmtDNA animals (Fig. 5E, F) and *pdr-1(mut);*+/ΔmtDNA (Fig. 5G, H). While mtDNA levels, including both ΔmtDNA and +mtDNA, were ~1.5-fold higher in the gonad vs. the soma of *pdr-1(mut)* animals (Wilcoxon Mann-Whitney rank sum test, *P* < 0.05; Fig. 5G, H), similar to wild type animals (Fig. 4F, G). We observed that *fzo-1(mut);pdr-1(mut);*+/ΔmtDNA animals displayed similar mtDNA levels in the gonad compared to soma, for both truncated and intact mtDNA molecules (Fig. 5E, F). On top of the selection against ΔmtDNA, *fzo-1(mut);pdr-1(mut);*+/ΔmtDNA double mutant displayed a reduction in total mtDNA levels, again supporting epistasis. Taken together, these data suggest that disrupting parkin-mediated mitophagy increased the organismal selection in *fzo-1(mut);pdr-1(mut);*+/ΔmtDNA individuals, which is associated with reduced fitness.

## Discussion

**Fig. 6:**
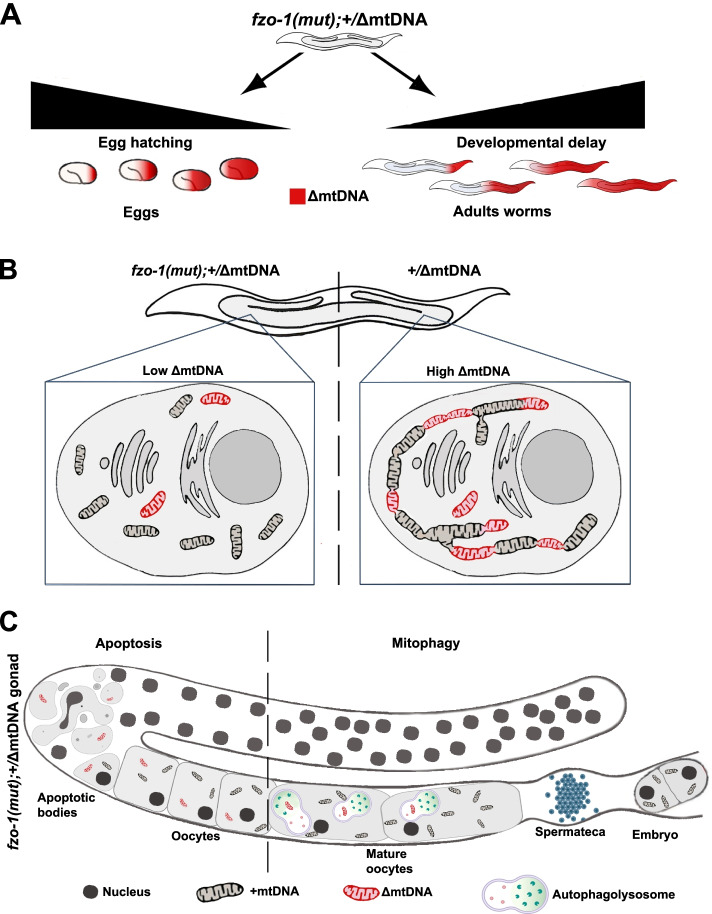
A model depicting selective removal of ΔmtDNA during development and gametogenesis of *C. elegans*. **A** Schematic representation—high ΔmtDNA levels (red) associate with reduced egg hatching and delayed development. **B** Schematic representation of mitochondria in the gonad, carrying intact +mtDNA (black) and ΔmtDNA (red). Mitochondria in *fzo-1(wt)* oocytes form an intracellular mitochondrial network suggested to enable mitochondrial functional complementation, hence allowing ΔmtDNA inheritance. In contrast, in *fzo-1(mut)* animals, the fusion machinery is compromised, mitochondria are fragmented, suggesting that ΔmtDNA molecules are ‘exposed’ and hence selectivity eliminated in the gonad of *fzo-1(mut)*;+/ΔmtDNA. **C** Two possible non-mutually exclusive mechanisms can explain the selective removal of ΔmtDNA molecules in the gonad of *fzo-1(mut)*;+/ΔmtDNA. Signals from mitochondria are suggested to trigger programmed cell death of germ cells when the transition from a globular to a tubular organization is disrupted, as in *fzo-1(mut)* (left). Sperm-derived signals are suggested to trigger lysosome acidification in mature oocytes, which can activate mitophagy (right). These processes can selectively impact the removal of mitochondria with high levels of ΔmtDNA, respectively, in *fzo-1(mut)* animals. The figure was created in part using BioRender.com

Our cross-generational analyses revealed complete loss of heteroplasmic deleterious mtDNA deletions when mitochondrial fusion is compromised. This demonstrated that heteroplasmy of deleterious mtDNA molecules could not be tolerated unless in the presence of a functional compensatory mechanism inherent to the mitochondrial network and the mitochondrial quality control machinery [14–16, 22, 23]. What drives this selection? It was previously found that *fzo-1* mutation impacted the developmental pace of the worms [35–37]. Nevertheless, we show that introducing heteroplasmic mtDNA deletions strongly aggravated these phenotypes, leading to a sharp decline in survival and fecundity. This was manifested by increased embryonic lethality and delayed or even arrested larval development of worms with high levels of deleterious heteroplasmic mtDNAs (Fig. 6A). This strong selective pressure also positively correlated with the decline in heteroplasmic deletion levels across generations. Since the loss of ΔmtDNA molecules is associated with the improved health condition of the worms within 3–4 generations, mitochondrial fusion is likely critical for tolerating ΔmtDNA molecules to maintain fitness.

In parallel, our discovery that the levels of heteroplasmic deletions are specifically reduced in the gonad when the fusion machinery is impaired (Fig. 6B) is consistent with previous findings of selective forces in the human germline [4, 5, 12]. Moreover, in *Drosophila*, germline selection of heteroplasmic mutations was directly observed in response to compromised fusion and quality control machinery [12, 21, 22]. We found that disrupting mitophagy in addition to mitochondrial fusion (*fzo-1(mut);pdr-1(mut)*) in the presence of ΔmtDNA sharply increased lethality already in G1 animals (reflected by the deviation from Mendelian ratios). Likewise, we found that disrupting Parkin-mediated mitophagy did not block germline selection, affecting the specific removal of mtDNA. These findings agree with germline selection in *Drosophila*, where downregulation of mitophagy factors, BNIP3 and Atg1, significantly blocked selection against fragmented mitochondria in germline cysts [22]. We, therefore, propose that selection against deleterious mtDNA molecules across generations affects the fitness of the organism, at two levels, namely during embryogenesis and larvae development as well as during oogenesis (Fig. 6A, B).

Two quality control processes were previously suggested to impact germline health and may contribute to selection against mutated mtDNA in *fzo-1(mut)* animals. Firstly, increased apoptosis was observed upon disruption of mitochondrial transition from a globular to a tubular organization in *C. elegans* oogenesis [38]. This may contribute to selective mitochondrial removal since selective export of mitochondria from germ cells undergoing apoptosis was observed [39]. Secondly, sperm-derived signals were found to induce lysosome acidification in mature oocytes prior to fertilization [40] and could promote mitophagy activation of fragmented mitochondria in *fzo-1(mut)* animals (Fig. 6C). Compromising either of these two processes led to reduced brood size. Therefore, we argue that the interaction between mitochondrial fusion and quality control machinery is not only critical to cope with ΔmtDNA within the cell but is essential for the organism’s fecundity, development, and survival across generations. Other selection mechanisms, such as selective replication, observed in *Drosophila* [12], or homologous recombination of a deletion and a corresponding duplication (in case of “triplasmy”) [41] could also contribute to selection against mutated mtDNA in *C. elegans*. Our results suggest that the latter explanations are less likely for the heteroplasmic strains described in this study.

Our analysis of three different mtDNA deletions in *C. elegans* revealed significant differences in the pace of their loss and levels of phenotypic severity when grown in the presence of a *fzo-1* mutant. Indeed, these three deletions differ in size and encompass different sets of mtDNA genes and tRNAs, suggesting that the fusion machinery is sensitive to differential severity of the phenotypic impact of heteroplasmic mutations. This finding is in line with differences in the penetrance of disease-causing mutations, which range between 60 and 80%, depending on the symptoms [9]. Nevertheless, the question about the functional importance of certain mtDNA regions versus others remains open. This calls for a screen of mtDNA mutants that will systematically enable assessing the sensitivity of the mitochondrial quality control and fusion machinery in differentiating the phenotypic impact of a variety of mutations, locations, and sizes.

If mitochondrial fusion is indeed important for modulating the inheritance of mtDNA heteroplasmy, one could anticipate that dysfunctional mitochondrial fusion, such as in the case of Charcot Marie Tooth type 2A (CMT2A) patients, will affect patterns of heteroplasmy. Our deep mtDNA sequencing analysis of three CMT2A pedigrees lends first clues that this might be the case [42]. Whereas two of the pedigrees did not reveal any potentially functional mtDNA mutations or deletions, we found that the levels of a potentially functional mtDNA mutation in a patient were notably lower than her healthy maternal relatives. We note that these results are in line with the observations in worms, supporting our working hypothesis that the fusion machinery modulates the levels of deleterious mtDNA heteroplasmy across generations to allow tolerance and survival. However, there are two mitofusin genes (MFN1 and MFN2) in humans, and MFN2 as well as DRP1 (inner membrane fusion) also function as tethers at mitochondria-associated ER membranes [43, 44] and could impact Parkin-mediated mitochondrial quality control [34, 45, 46]. Future collection of a larger number of CMT2A pedigrees is required to draw clearer conclusions.

The complete loss of ΔmtDNA across *C. elegans* generations underlines the fusion machinery as an attractive candidate target for future treatment of mitochondrial disorders. For example, the activity of protein quality control systems, including this machinery, declines during the aging of the individual [47, 48], and the levels/repertoire of mtDNA heteroplasmic deletions increase in tissues from aged individuals [49]. It would, therefore, be of great interest to assess the importance of such three-way interaction (i.e., mitochondrial fusion-mitochondrial quality control and patterns of heteroplasmy) to the tendency to develop age-associated diseases.

## Conclusions

Here, by manipulating the fusion machinery in *C. elegans*, we demonstrated that *fzo-1* (mitofusin) is a key modulator of mtDNA heteroplasmy while showing its impact on the transmission of heteroplasmic deletions across generations in living animals. Firstly, we discovered that a *fzo-1(mut)* led to the complete loss of three different mtDNA deletions, separately. These findings provide experimental support for the hypothesis that functional complementation among mitochondria in the intracellular network likely enables the survival and prevalence of deleterious heteroplasmic mtDNA mutations in the population [25, 26]. Secondly, we found that the *fzo-1(mut)* was differentially sensitive to the size/nucleotide positions of the different mtDNA deletions. Third, *fzo-1(mut)*;*pdr-1(mut)* double mutants selected against the survival of animals with heteroplasmic ΔmtDNA deletion and accelerated the loss of ΔmtDNA molecules across generations. Taken together, our results demonstrate the importance of cross-talk between mitochondrial fusion and the mitochondrial quality control machinery in protecting living animals from the adverse impact of inheriting deleterious mtDNA molecules.

## Methods

### Nematodes and growth conditions

A list of strains used in this work and name abbreviations are found in Additional file 1: Table S4. All strains were outcrosses to our N2 stock at least four times. Nematodes were grown on Nematode Growth Medium (NGM) plates seeded with the *Escherichia coli* OP50-1 strain at 15 °C.

### Statistical analyses

To test the null hypothesis that the heteroplasmic deletions reduce the fitness of WT (Additional file 1: Figs. S2B and S3E), *fzo-1(mut)* (Fig. 1B, C and Fig. 2C) or *fzo-1(mut);pdr-1(mut);* (Additional file 1: Fig. S4A-C) as compared to wild type or *fzo-1(mut)* animals, we used one-way analysis of variance (ANOVA) followed by a Tukey’s post hoc test. We used the same test to compare the levels of ΔmtDNA, +mtDNA, and TMRE staining in *fzo-1(mut)* and *fzo-1(wt)* strains (Fig. 2F, Fig. 4A, B, and Additional file 1: S1B). Data are presented as bar graphs showing means ±1 standard error of the mean (1SE). To compare the mtDNA (ΔmtDNA and/or +mtDNA) levels between two conditions and assess statistical significance (Fig. 4C–G, Fig. 5D–H, and Additional file 1: S3C-D), we used the Wilcoxon Mann-Whitney rank sum test. Data are presented as scatter dot plots showing points for data or bar graphs showing means ± 1SE. To test whether heteroplasmic progeny deviated from the expected Mendelian ratio, we used *χ*^2^ goodness of fit test, data are presented in Additional file 1: Table S1. To examine differences in developmental rate across generations of *fzo-1* mutant (Figs. 2B and 5B) or wild type animals (Additional file 1: Figs. S2A and S3F), we used Cox proportional-hazards regressions (Additional file 1: Table S2). To control for the dependency of individuals within biological repeats, a robust jackknife variance estimator grouped by observations per experimental plate was used. Data points showing the percent of total animals that reached adulthood within the experimental time (11 days) are presented as line graphs. To test for changes in heteroplasmic deletions (Fig. 2D, E, Fig. 3A, B, Fig. 5C, and Additional file 1: S3H-I, S4D) or +mtDNA (Additional file 1: Fig. S2C-D) levels across generations of *fzo-1* mutant (Fig. 2D, Fig. 3A, B, Fig. 5C, and Additional file 1: S2C) or wild type animals (Fig. 2E and Additional file 1: S2D, S3H-I, and S4D), we used fractional regressions with logit link function (Additional file 1: Table S3). To compare the change in heteroplasmic deletion levels across generations in the different genetic backgrounds (Fig. 3C), we used a fractional regression with logit link function followed by within generation pairwise comparisons (Additional file 1: Table S3). To control for the dependency of individuals within biological repeats, a robust variance estimator grouped by observations per experimental plate was used. Odds ratios were calculated to determine the likelihood of heteroplasmy in a given generation. Data are presented in box plot representation: center line, median; box limits, upper and lower quartiles; whiskers, minimum and maximum; points, data. The numbers of biological repeats (*N*) and individuals (*n*) in each condition tested are noted in the figure legends (Figs. 1, 2, 3, and 5 and Additional file 1: S1-S4).

### Single worm genotyping

Animal genotype was determined using a single worm PCR Phire Animal Tissue Direct PCR Kit (Thermo Scientific) with primers to detect *fzo-1* or *pdr-1* deletions. The list of PCR primers is found in Additional file 1: Table S5. The resultant amplification products were visualized by gel electrophoresis to determine the genotype.

### Establishing and maintaining fzo-1 heterozygotes heteroplasmic lines

Mutant *fzo-1(tm1133)* animals (strain CU5991) are very poor in mating and, therefore, were first crossed with males expressing a yellow fluorescent protein marker (*unc-54p::YFP*). Heteroplasmic hermaphrodites (*uaDf5/+, bguDf1/+* or *bguDf2/+*) were then crossed with *fzo-1(tm1133);unc-54p::YFP* heterozygote males to ensure maternal inheritance of mtDNA deletions (Fig. 1A). Heteroplasmic (*uaDf5/+, bguDf1/+* or *bguDf2/+*) animals that were heterozygotes for *fzo-1(tm1133)*, *fzo-1(ht),* were identified and maintained using single worm genotyping, establishing independent heteroplasmic lines carrying *fzo-1(ht)*.

Mutant *pdr-1(gk448)* animals (strain VC1024) were first crossed with heteroplasmic hermaphrodites *(uaDf5/+)* and with *fzo-1(tm1133)* to establish double mutant strains. *fzo-1(tm1133);pdr-1(gk448)* double mutants were crossed with *unc-54p::YFP* (marker); then, the F1 heterozygote males, *fzo-1(tm1133);pdr-1(gk448);unc-54p::YFP*, were crossed with heteroplasmic hermaphrodites, ΔmtDNA;*pdr-1(gk448)* to ensure maternal inheritance of *uaDf5* (Fig. 5A). Heteroplasmic (*uaDf5/+*) animals that were homozygous to *pdr-1(gk448), pdr-1(mut)*, and heterozygotes for *fzo-1(tm1133)*, were identified using PCR genotyping. *fzo-1(ht)* were then maintained using single worm genotyping to establish a heteroplasmic line carrying *fzo-1(ht);pdr-1(mut)*.

### Monitoring animals across generations

Single animals from the heterozygotes heteroplasmic lines (*uaDf5/+, bguDf1/+,* or *bguDf2/+*) were isolated, allowed to lay eggs, and genotyped using a single worm PCR for *fzo-1*. The progeny of *fzo-1(ht)* was again isolated, allowed to lay eggs, and screened to identify mutant or wild type *fzo-1* animals (G1m and G1wt, respectively). Heterozygous progeny was maintained to generate G1. The progeny of mutant or wild type animals (G2m and G2wt, respectively) was then monitored and/or isolated and allowed to lay eggs. This was repeated over several generations (G2m-G4m and G2wt-G4wt, respectively; Fig. 2A).

To test for residual ΔmtDNA (*uaDf5*, *bguDf1*, or *bguDf2*), G4m hermaphrodites were crossed with wild type males; the progeny was allowed to self-propagate, isolated, allowed to lay eggs, and genotyped. Heteroplasmy levels in *fzo-1(wt)* animals (Gm→Gwt) were then examined.

### Embryo hatching

Gravid animals were moved to a fresh plate for 2–12 h and then removed from the plates. Hatching was examined after 48 h. The numbers of biological repeats (*N*) and individuals examined (*n*) in each condition tested are noted in the figure legends (Fig. 1B, Fig. 2C and Additional file 1: S2B, S3E and S4A). Individual data values are included in Additional file 2.

### Developmental timing

Single embryos were placed on fresh plates and allowed to grow at 15 °C. The animals’ developmental stage was examined every day, and the number of animals reaching reproductive adulthood on each day was recorded. Developmentally arrested animals that did not reach adulthood in over 11 days were excluded. The numbers of biological repeats (*N*) and individuals examined (*n*) in each condition tested are noted in the Figure legends (Fig. 1C, Fig. 2B, Fig. 5B and Additional file 1: S2A, S3F and S4B-C). Individual data values are included in Additional file 2.

### Mitochondria staining and membrane potential assay

Age-synchronized adults were placed on NGM plates seeded with the *E. coli* OP50-1 and containing 100 *μ*M MitoTracker Deep Red FM (Thermofisher) or 100 *μ*M TMRE (tetramethylrhodamine, ethyl ester) (Biotum). The animals were kept on the plates for 24 h in the dark and then recovered on regular plates for 2 h. Animals were then fixed with 4% paraformaldehyde and imaged using a LEICA DM5500 B epifluorescence microscope. MitoTracker was imaged using a × 40 or a × 60 numerical aperture objective with a 633-nm line for excitation. TMRE was imaged using a × 10 numerical aperture objective with a 549-nm line for excitation. TMRE staining was quantified using CellProfiler cell image analysis software. The numbers of individuals examined (*n*) in each condition tested are noted in the figure legend of Additional file 1: S1B. Individual data values are included in Additional file 2.

### DNA purification and extraction

Total DNA was extracted using a QuickExtract kit (Lucigen). Unless otherwise indicated, DNA was extracted from a single worm. When populations were examined, ~ 5 animals were collected. For embryos, DNA was extracted from 15 to 30 embryos. For gonad-soma analysis, gonads were dissected from 5 to 10 animals per biological repeat. DNA was extracted separately from the gonads and soma.

### Quantification of mtDNA copy numbers

mtDNA levels were measured by qPCR performed on a C1000 Thermal Cycler (Bio-Rad) with KAPA SYBRFAST qPCR Master Mix (KAPA Biosystems). Analysis of the results was performed using CFX Manager software (Bio-Rad). To quantify the different mtDNA molecules, three sets of primers were used for truncated (ΔmtDNA), intact (+mtDNA), and total mtDNA molecules for each of the three deletions examined (Additional file 1: Table S5). ΔmtDNA levels were determined using primers located in the boundaries of the deletions and thus amplified only from the truncated copies. +mtDNA levels were determined using one primer located within the deletion and a second primer located outside of the deletion and thus amplified only from the intact copies. Total mtDNA levels were determined using primers located outside of the deletion area. For each sample, the average *C*_T_ (threshold cycle) of triplicate values obtained for these mtDNA molecules was normalized to a nuclear DNA marker using the 2^−ΔΔC^_T_ method [50]. Truncated/total or intact/total ratio was defined as the ratio of the normalized *C*_T_ values of truncated to total mtDNA for a given animal or strain. The numbers of biological repeats (*N*) and individuals examined (*n*) in each condition tested are noted in the figure legends (Figs. 2D–F, 3A, B, 4A–G and 5C–H and Additional file 1: S2C-D, S3C-D, S3H-I and S4D). Individual data values are included in Additional file 2.

### Reanalysis of whole-genome sequencing

The occurrence of deletions and duplications in the mtDNA was assessed by analyzing whole-genome sequencing reads from the NCBI’s Sequence Read Archive (SRA) database, corresponding to two of the strains used in this study: SRR801606 - SRR801609 for strain VC40128, and SRR793379 - SRR793382 for strain VC20469 [30]. Reads were downloaded, trimmed according to quality scores (default parameters), and filtered for excluding read-through adapters sequences. The processed reads were mapped against the entire genome of the N2 wild type strain to exclude contamination of nuclear mitochondrial DNA (NUMTs) in our results. N2 genome information was downloaded from NCBI’s RefSeq database (accession numbers: Chr_1 - NC_003279.8, Chr_2 - NC_003280.10, Chr_3 - NC_003281.10, Chr_4 - NC_003282.8, Chr_5 - NC_003283.11, Chr_X - NC_003284.9, and Chr_M - NC_001328.1). We used a genome browser to visualize reads coverage and detect the previously reported deletions in the examined strains [29]. Deletions and duplications were determined by two parameters; a relative change in sequencing coverage and the mapping of broken read pairs at the edges of the deletions (i.e., new sites formed by the deletion or duplication). The algorithm would identify pairs as broken if the observed distance post mapping between the pairs was significantly larger than the expected distance, according to the size selection procedure during library preparation. Analyses described above were performed using CLC Genomics workbench 20, QIAGEN.

### Gonad dissection

G2 wild type or mutant animals were placed in a drop of ultra-pure water on a coverslip slide, and a 25-gauge needle was used to remove the gonads from the body of the animals. Gonads or the remaining carcasses were then transferred to a DNA extraction buffer.

## Supplementary Information

**Additional file 1: Table S1.** Genotypes distribution of F2 progeny in different heteroplasmic strains. **Table S2.** Cox proportional-hazards regression analyses. **Table S3.** Fractional regression analyses. **Table S4.** A list of *C. elegans* strains used in this study. **Table S5.** A list of primers used in this study. **Figure S1.** Characterization of +/ΔmtDNA animals. **Figure S2.** Characterization of *fzo-1(wt);+/ΔmtDNA;* animals. **Figure S3.** Characterization of the 1kbΔmtDNA and 4kbΔmtDNA animals. **Figure S4.** Characterization of *fzo-1(wt);pdr-1(mut);+/ΔmtDNA* animals.

**Additional file 2.** Individual data values. Spreadsheets of numerical data for Figures 1, 2, 3, 4 and 5 and Supplementary Figures S1-S4.

## Data Availability

All data generated or analyzed during this study are included in this published article and its supplementary information files. Individual data values are included in Additional file 2. The sequencing datasets analyzed in the current study are available in the NCBI Sequence Read Archive repository accession number SRP018046 [30], http://www.ncbi.nlm.nih.gov/sra.
