# Supplementary material for: Mutant C. elegans mitofusin leads to selective removal of mtDNA heteroplasmic deletions across generations to maintain fitness
Source: BMC Biol. 2022 Feb 9;20:40. doi: 10.1186/s12915-022-01241-2 (PMC8829988; doi:10.1186/s12915-022-01241-2)
Supplement: Supplementary file 1 — Additional file 1: Table S1. Genotypes distribution of F2 progeny in different heteroplasmic strains. Table S2. Cox proportional-hazards regression analyses. Table S3. Fractional regression analyses. Table S4. A list of C. elegans strains used in this study. Table S5. A list of primers used in this study. Figure S1. Characterization of +/ΔmtDNA animals. Figure S2. Characterization of fzo-1(wt);+/ΔmtDNA; animals. Figure S3. Characterization of the 1kbΔmtDNA and 4kbΔmtDNA animals. Figure S4. Characterization of fzo-1(wt);pdr-1(mut);+/ΔmtDNA animals. [file 12915_2022_1241_MOESM1_ESM.pdf]

Supplementary Information

Mutant *C. elegans* mitofusin leads to selective removal of mtDNA heteroplasmic deletions across generations to maintain fitness

Meshnik *et al.*

Supplementary Tables S1-S5

Supplementary Figures S1-S4

Supplementary Tables

Table S1: Genotypes distribution of F2 progeny in different heteroplasmic strains.

Genotype	Progeny (#)	Progeny (%)	χ^2 test (N)
fzo-1(wt);ΔmtDNA	38	25.3	0.96 (150)
fzo-1(ht);ΔmtDNA	73	48.7	
fzo-1(mut);ΔmtDNA	39	26	
fzo-1(wt);1kbΔmtDNA	61	33.3	0.14 (183)
fzo-1(ht);1kbΔmtDNA	79	43.2	
fzo-1(mut);1kbΔmtDNA	43	23.5	
fzo-1(wt);4kbΔmtDNA	48	34.3	P<0.001 (140)
fzo-1(ht);4kbΔmtDNA	83	59.3	
fzo-1(mut);4kbΔmtDNA	9	6.4	
fzo-1(wt);pdr-1(mut);ΔmtDNA	70	35.2	P<0.001 (199)
fzo-1(ht);pdr-1(mut);ΔmtDNA	111	55.8	
fzo-1(mut);pdr-1(mut);ΔmtDNA	18	9	

Table S2: Cox proportional-hazards regression analyses.

Fig.	Genotype	Comparing	Coef. (β)	SE	Z	*P*	**Model *Stat.*** Wald test (Degrees of freedom) *P* value
2B	*fzo-1(mut)*; +/ΔmtDNA	G1m-G2m	-1.864	0.194	-14.898	<0.001	224 (3) P<0.001
		G1m-G3m	-0.466	0.176	-4.024	<0.001	
		G1m-G4m	0.056	0.190	0.319	0.750	
5B	*fzo-1(mut)*; *pdr-1(mut)* +/ΔmtDNA	G30m-G2m	-1.047	0.310	-3.37	<0.001	25.4 (3) *P*<0.001
		G30m-G3m	-0.635	0.185	-3.43	<0.001	
		G30m-G4m	-0.558	0.322	-1.73	0.084	
S2A	*fzo-1(wt)*; +/ΔmtDNA	G1wt-G2wt	0.467	0.409	1.14	0.250	19.5 (3) *P*<0.001
		G1wt-G3wt	0.099	0.397	0.25	0.800	
		G1wt-G4wt	0.595	0.398	1.49	0.140	
S3F	+/1kb ΔmtDNA	WT-1kb ΔmtDNA	-0.113	0.051	-2.19	0.028	4.88 (2) *P*<0.087
	+/4kb ΔmtDNA	WT-4kb ΔmtDNA	-0.083	0.064	-1.28	0.200	

Summary results of Cox proportional regressions testing for cross-generational changes in the rate at which *C. elegans* strains, carrying different heteroplasmic mtDNA deletions, turn into adults. To control for the dependency of individuals within biological repeats, a robust jackknife variance estimator grouped by observations per experimental plate was used.

Table S3: Fractional regression analyses.

Fig.	Genotypes	Comparing	Coef. (β)	SE	Z	*P*	Model *Stat.* Wald test (Degrees of freedom); *P*; Pseudo R^2
2D	+/ΔmtDNA (+/3kb)<br><br>*fzo-1(mut)*; +/ΔmtDNA (+/3kb)	+/3kb-G1ht	1.325	0.455	2.91	<0.004	2231.62 (6)<br>*P*<0.001<br>R^2=0.428
		+/3kb-G1m	-0.306	0.472	-0.06	0.948	
		+/3kb-G2m	-2.533	0.564	-4.49	<0.001	
		+/3kb-G3m	-7.294	0.679	-10.74	<0.001	
		+/3kb-G4m	-8.864	0.812	-10.92	<0.001	
		+/3kb-Gm->wt	-9.574	0.488	-19.61	<0.001	
		Intercept	-0.649	0.438	-0.15	0.882	
2E	*fzo-1(wt)*; +/ΔmtDNA (+/3kb)	G1wt-G2wt	-0.246	0.340	-0.72	0.468	0.99 (3)<br>*P*=0.803<br>R^2=0.003
		G1wt-G3wt	-0.378	0.386	-0.98	0.327	
		G1wt-G4wt	-0.192	0.431	-0.44	0.657	
		Intercept	1.553	0.282	5.50	<0.001	
3A	*fzo-1(mut)*; +/ΔmtDNA (+/1kb)	+/1kb-G1ht	0.613	0.076	8.09	<0.001	57127.21 (6)<br>*P*<0.001<br>R^2=0.419
		+/1kb-G1m	-0.933	0.242	-3.85	<0.001	
		+/1kb-G2m	-4.021	0.419	-9.61	<0.001	
		+/1kb-G3m	-8.283	0.224	-36.92	<0.001	
		+/1kb-G4m	-9.367	0.054	-171.25	<0.001	
		+/1kb-Gm->wt	-8.779	0.139	-63.12	<0.001	
		Intercept	-0.418	0.053	-7.95	<0.001	
3B	*fzo-1(mut)*; +/ΔmtDNA (+/4kb)	+/4kb-G1ht	0.195	0.266	0.73	0.464	11295.45 (6)<br>*P*<0.001<br>R^2=0.552
		+/4kb-G1m	-1.631	0.256	-6.37	<0.001	
		+/4kb-G2m	-4.166	0.211	-19.75	<0.001	
		+/4kb-G3m	-6.932	0.208	-33.28	<0.001	
		+/4kb-G4m	-7.816	0.222	-35.22	<0.001	
		+/4kb-Gm->wt	-8.953	0.294	-30.46	<0.001	
		Intercept	1.224	0.208	5.87	<0.001	
3C	+/ΔmtDNA: 1kb;3kb;4kb 1Δ;3Δ;4Δ<br><br>*fzo-1(mut)*; +/ΔmtDNA: 1kb;3kb;4kb G_{1-4}<br><br>+/Δ all parental truncated strains	3Δ;G_{1-4}-1Δ;G_{1-4}	-0.353	0.438	-0.81	0.420	95626.48(14)<br>*P*<0.001<br>R^2=0.405
		3Δ;G_{1-4}-4Δ;G_{1-4}	1.288	0.480	2.68	0.007	
		+/Δ- allG1m	-0.031	0.469	-0.07	0.888	
		+/Δ- allG2m	-2.533	0.560	-4.52	<0.001	
		+/Δ- allG3m	-7.294	0.674	-10.82	<0.001	
		+/Δ- allG4m	-8.864	0.806	-10.99	<0.001	
		3kb- 1kbG1	-0.902	0.527	-1.71	0.087	
		3kb- 1kbG2	-1.487	0.696	-2.14	0.033	
		3kb- 1kbG3	-0.989	0.710	-1.39	0.163	
		3kb- 1kbG4	-0.503	0.808	-0.62	0.536	
		3kb- 4kbG1	-1.600	0.532	-3.01	0.003	
		3kb- 4kbG2	-1.632	0.597	-2.73	0.006	
		3kb- 4kbG3	0.363	0.704	0.51	0.607	
		3kb- 4kbG4	1.048	0.835	1.26	0.209	
		Intercept	-0.065	0.435	-0.15	0.881	
5C	*fzo-1(mut)*; *pdr-1(mut)* +/ΔmtDNA: (+/3kb)	G1ht-G1m	-1.863	0.577	-3.23	0.001	8246.02 (4)<br>*P*<0.001<br>R^2=0.402
		G1ht-G2m	-4.164	0.521	-7.98	<0.001	
		G1ht-G3m	-8.559	0.721	-11.87	<0.001	
		G1ht-G4m	-9.902	0.519	-19.09	<0.001	
		Intercept	0.388	0.518	0.75	0.454	

5C vs. 2D	*fzo-1(mut)*; +/ΔmtDNA: (+/3kb)*;*Gf1-4 *fzo-1(mut)*; *pdr-1(mut)* +/ΔmtDNA: (+/3kb); Gfp1-4	Gf1-4- Gfp1-4	-1.380	0.308	-4.48	<0.001	10424.62 (7) P<0.001 R^2=0.371
		G1f;fp- G2f;fp	-2.503	0.398	-6.29	<0.001	
		G1f;fp- G3f;fp	-7.264	0.549	-13.22	<0.001	
		G1f;fp- G4f;fp	-8.834	0.756	-11.69	<0.001	
		G1f- G2fp	0.202	0.475	0.43	0.670	
		G1f- G3fp	0.568	0.782	0.73	0.468	
		G1f- G4fp	0.795	0.797	1.00	0.319	
		Intercept	-0.095	0.178	-0.54	0.592	
S2C (^)	+/ΔmtDNA (+/3kb) *fzo-1(mut)*; +/ΔmtDNA (+/3kb)	+/3kb-G1ht	-0.964	0.382	-2.52	0.012	539.60 (6) P<0.001 R^2=0.195
		+/3kb-G1m	-0.172	0.372	-0.46	0.643	
		+/3kb-G2m	1.413	0.400	3.53	<0.001	
		+/3kb-G3m	1.858	0.385	4.82	<0.001	
		+/3kb-G4m	2.432	0.449	6.16	<0.001	
		+/3kb-Gm->wt	2.557	0.362	5.69	<0.001	
		Intercept	0.201	0.362	0.56	0.579	
S2D (^)	*fzo-1(wt)*; +/ΔmtDNA (+/3kb)	G1wt-G2wt	0.026	0.175	0.15	0.882	0.79 (3) P=0.852 R^2=0.002
		G1wt-G3wt	0.199	0.267	0.75	0.456	
		G1wt-G4wt	-0.026	0.214	-0.12	0.905	
		Intercept	-1.040	0.156	-6.46	<0.001	
S3H	*fzo-1(wt)*; +/ΔmtDNA (+/1kb)	G1wt-G2wt	0.380	0.170	2.23	0.026	5.70 (3) P=0.127 R^2=0.003
		G1wt-G3wt	0.255	0.133	1.91	0.056	
		G1wt-G4wt	0.184	0.142	1.30	0.194	
		Intercept	1.517	0.117	12.99	<0.001	
S3I	*fzo-1(wt)*; +/ΔmtDNA (+/4kb)	G1wt-G2wt	-0.172	0.178	-0.97	0.333	5.87 (3) P=0.118 R^2=0.003
		G1wt-G3wt	0.083	0.158	0.52	0.600	
		G1wt-G4wt	0.169	0.230	0.73	0.462	
		Intercept	-0.210	0.148	-1.42	0.157	
S4D	*fzo-1(wt)*; *pdr-1(mut)* +/ΔmtDNA: (+/3kb)	G1wt-G2wt	0.358	0.258	1.38	0.167	12.24 (3) P=0.007 R^2=0.006
		G1wt-G3wt	-0.014	0.232	-0.06	0.952	
		G1wt-G4wt	-0.176	0.229	-0.77	0.444	
		Intercept	1.076	0.219	4.92	<0.001	

Summary results of fractional regressions testing for cross-generational changes in the levels of ΔmtDNA or mtDNA (marked with ^) in *C. elegans* strains, carrying different heteroplasmic mtDNA deletions as noted. To control for the dependency of individuals within biological repeats, a robust variance estimator grouped by observations per experimental plate was used.

Table S4: A list of *C. elegans* strains used in this study.

Strain	Abbreviation	Nuclear genotype	Mitochondrial genotype
**N2**	wild type (WT)	---	--
**AM134**	Q0	*unc-54p::Q0::YFP*	--
**ABZ271***	*fzo-1*	*fzo-1(tm1133)*<br>derived from CU5991	--
**ABZ283***	*pdr-1*	*pdr-1(gk448)*<br>derived from VC1024	--
**ABZ270***	ΔmtDNA	--	uaDf5/+<br>derived from LB138
	G1ht	*fzo-1 +/-*	uaDf5/+
**ABZ272**	*fzo-1(mut);ΔmtDNA*	*fzo-1(tm1133)*	uaDf5/+**
**ABZ273**	*fzo-1(wt);ΔmtDNA*	*fzo-1(+)*	uaDf5/+
**ABZ274**	G4mut->G6wt	*fzo-1(+)*	uaDf5/+**
**ABZ275***	1kbΔmtDNA	--	*bguDf1*/+<br>derived from VC41028
	G1ht 1kbΔmtDNA	*fzo-1 +/-*	*bguDf1*/+
**ABZ276**	*fzo-1(mut);1kbΔmtDNA*	*fzo-1(tm1133)*	*bguDf1*/+**
**ABZ277**	*fzo-1(wt);1kbΔmtDNA*	*fzo-1(+)*	*bguDf1*/+
**ABZ278**	G4mut->G6wt	*fzo-1(+)*	*bguDf1*/+**
**ABZ279***	4kbΔmtDNA	--	*bguDf2*/+<br>derived from VC20469
	G1ht 4kbΔmtDNA	*fzo-1 +/-*	*bguDf2*/+
**ABZ280**	*fzo-1(mut);4kbΔmtDNA*	*fzo-1(tm1133)*	*bguDf2*/+**
**ABZ281**	*fzo-1(wt);4kbΔmtDNA*	*fzo-1(+)*	*bguDf2*/+
**ABZ282**	G4mut->G6wt	*fzo-1(+)*	*bguDf2*/+**
**ABZ285**	*pdr-1;*ΔmtDNA	*pdr-1(gk448)*	uaDf5/+
**ABZ284**	*fzo-1;pdr-1*	*fzo-1(tm1133);pdr-1(gk448)*	--
**ABZ286**	*fzo-1(mut);pdr-1(mut);*<br>*ΔmtDNA*	*fzo-1(tm1133);pdr-1(gk448)*	uaDf5/+**
**ABZ287**	*fzo-1(wt);pdr-1(mut);*<br>*ΔmtDNA*	*fzo-1(+);pdr-1(gk448)*	uaDf5/+

* Strains were outcrossed with our lab N2 stock at least four times
** ΔmtDNA levels were lost over generations
- CU5991, VC1024, and LB138 (after outcrossing ABZ271, ABZ283, and ABZ270, respectively) were provided by the *Caenorhabditis* Genetics Center,
- VC40128 and VC20469 (after outcrossing ABZ275 and ABZ279, respectively) were provided by the *C. elegans* Reverse Genetics Core Facility at the University of British Columbia.

Table S5: **A list of primers used in this study.**

Primer	Primer sequence
fzo-1	F-TCTGAGCACACTTCAAGCTC
	R- TGCTGCCGATAATGAACCAC
pdr-1	F-CGGTCGCTGTGAGTTTAGAA
	R-GGAGTACAGCATTCTTCGCA -
3kbΔmtDNA (ΔmtDNA)	F -TGAGACTTTTAATTATTTACATCCC
	R-CAGTGCATTGACCTAGTCATC-
1kbΔmtDNA	F-ATACTTTACCATTAAGGTCAGTAATTTCTA
	R- GTTGTTTCTCAATTAATAAAATTATAACCCC
4kbΔmtDNA	F-TCAAGGAGGATTGGCAGTTTGA
	R-ACCTCTAAAAACCGATAAACCAAAA
Intact (+)3kbΔmtDNA and total 4kbΔmtDNA	F -ATGGGATGTTGGTGACATTGC
	R-TGCTATTAACCTATCGGGCGTA
Intact (+)1kbΔmtDNA	F-ATACTTTACCATTAAGGTCAGTAATTTCTA
	R-TCACGCTACAGCAGCATAAAC
Intact (+)4kbΔmtDNA	F-TCAAGGAGGATTGGCAGTTTGA
	R-TCTAGTACCAACCATACCAGATCA
Total of 3bΔmtDNA and 1kbΔmtDNA	F-TCGGTGGTTTTGGTAACTGAT
	R- AAAGGTGGGTAGACTGTTCAC
Genomic DNA control	F-CTGGAAGAAGATAATTATTTTCC
	R-CTGTATTCTCCGGATTACGAG

Supplementary Figures

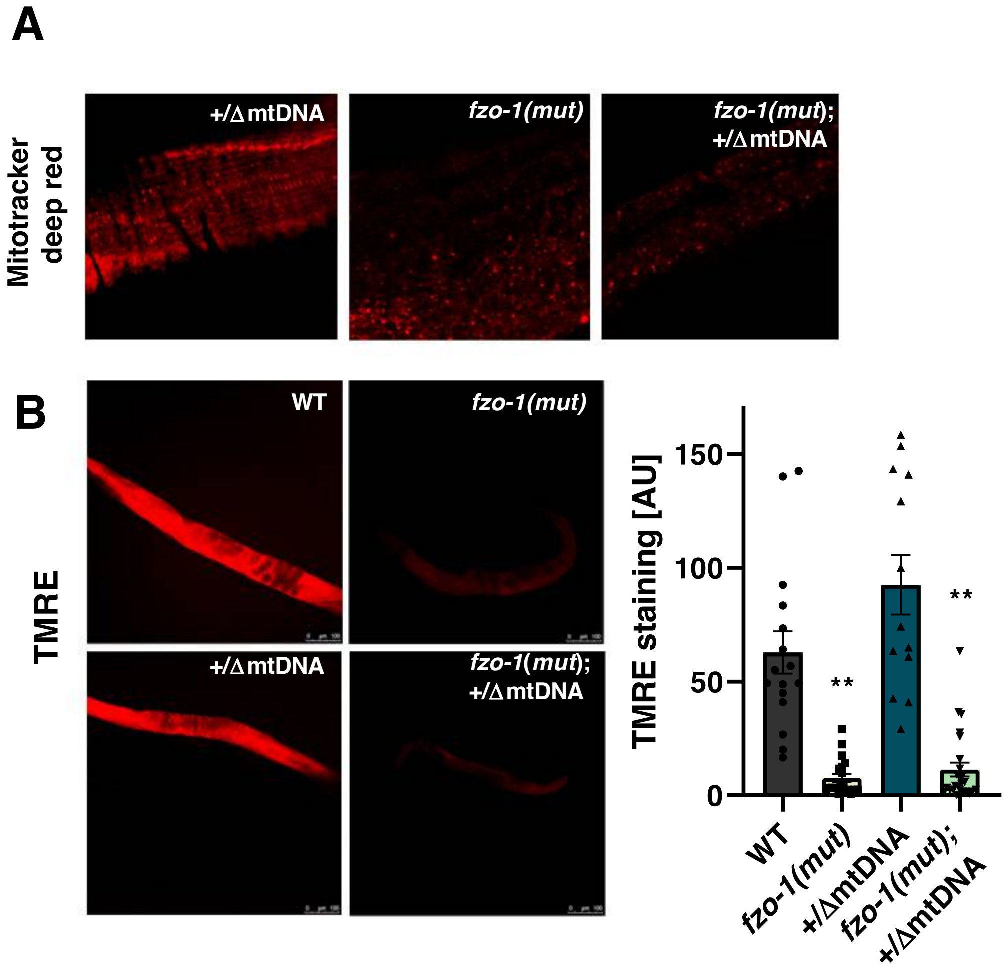

Figure S1: Characterization of +/ΔmtDNA animals.

(A) Confocal images of mitochondria in body-wall muscle cells. Age synchronized adults of the parental strains, *fzo-1(mut)* and +/ΔmtDNA and cross progeny +/ΔmtDNA;*fzo-1(mut)* stained MitoTracker Deep Red (24 hours) were washed, fixed and imaged.

(B) TMRE staining. Age synchronized adults of the parental strains, *fzo-1(mut)* and +/ΔmtDNA and cross progeny +/ΔmtDNA;*fzo-1(mut)* stained with TMRE (24 hours) were washed, fixed, and imaged (left). TMRE staining in individual worms was quantified using CellProfiler cell image analysis software (right; (N>3) wt n=16; *fzo-1(mut)* n=20; +/ΔmtDNA n=13, and *fzo-1(mut);*+/ΔmtDNA, n=25). Data are means ± 1 standard error of the mean (1SE). Data were analyzed using one-way ANOVA followed by a Tukey's *post-hoc* test, (**) denotes $P<0.001$. Individual data values are presented in Additional file 2.

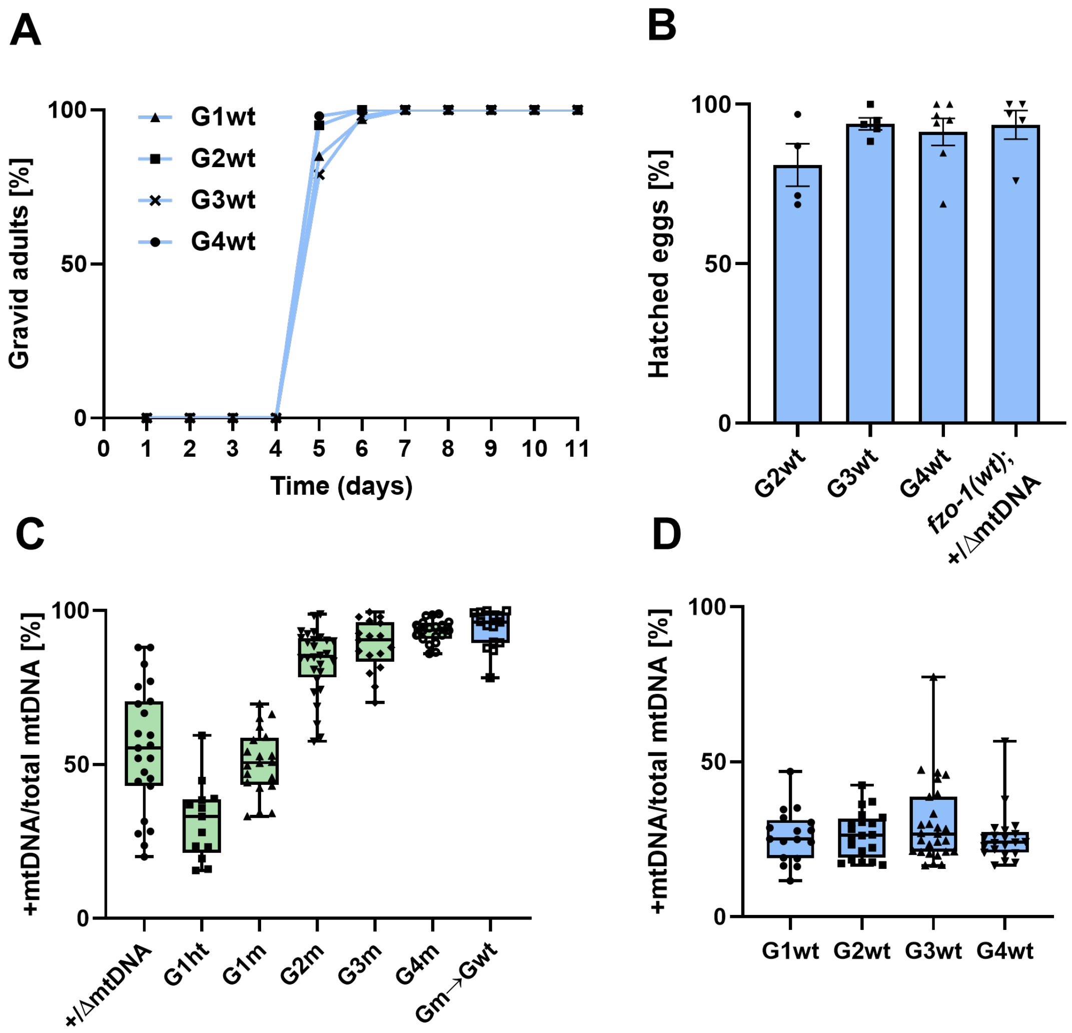

Figure S2: Characterization of *fzo-1(wt);*+/ΔmtDNA animals.

(A) The percent of gravid adults of *fzo-1(wt)*;+/ΔmtDNA wild type progeny across generations (G1wt-G4wt) at the indicated times after egg laying (N>3 biological repeats, G1wt N=4, n=29, G2wt N=5, n=245, G3wt N=3, n=124, and G4wt N=5, n=173). Data were analyzed using Cox proportional-hazards regression (Additional file 1: Table S2). No significant differences were observed between G1wt and G2wt-G4wt.

(B) The percent of hatched embryos of wild type *fzo-1(wt);*+/ΔmtDNA progeny across generations (G2wt N=4, n=104; G3wt N=5, n=104; and G4wt N=7, n=277) and the stable line (>20 generations) *fzo-1(wt);*+/ΔmtDNA (N=5 n=134). Data are means ± 1 standard error of the mean (1SE). Data were analyzed using one-way ANOVA followed by a Tukey's *post-hoc* test.

(C) Box plot showing the percent of +mtDNA (N>3 biological repeats) determined in individual animals of the parental heteroplasmic strain +/ΔmtDNA (n=23), the heteroplasmic *fzo-1(mut)* mutant cross-progeny strains (G1(ht) n=13, G1m-G4m n=20, 28, 17 and 19, respectively) and the progeny of G4m animals crossed with *fzo-1(wt)*, (Gm→Gwt; n=15). In the boxplot representation, center line, median; box limits, upper and

lower quartiles; whiskers, minimum and maximum; points, data. Data were analyzed using Fractional regression (Additional file 1: Table S3). +mtDNA levels of G2m-G4m and Gm->Gwt were significantly higher than in +/ΔmtDNA, (**) denotes *P*<0.001.

(D) Box plot showing the percent of +mtDNA (N>3 biological repeats) determined in individual animals of the *fzo-1(wt)* cross progeny strains (G1wt-G4wt n=17, 20, 27, and 21, respectively). In the boxplot representation, center line, median; box limits, upper and lower quartiles; whiskers, whiskers, minimum and maximum; points, data. Data were analyzed using Fractional regression (Additional file 1: Table S3). Differences in ΔmtDNA levels were not significant. Individual data values are presented in Additional file 2.

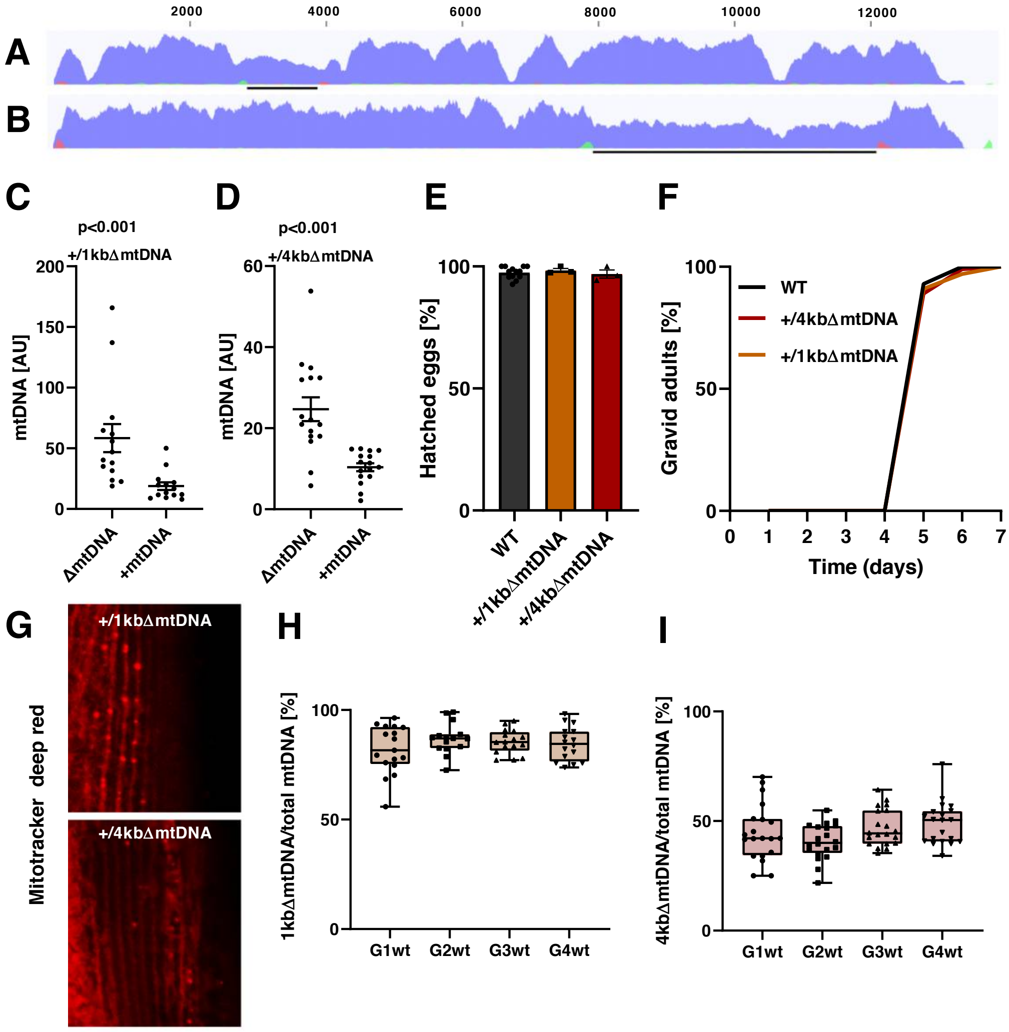

Figure S3: Characterization of the 1kbΔmtDNA and 4kbΔmtDNA animals.

(A-B) Genome Browser display showing the read coverage across the mitochondrial genome in the 1kbΔmtDNA (VC40128; A) and 4kbΔmtDNA (VC20469; B) strains. Underlines indicate the location of the truncations based on broken read pairs mapping.

(C-D) Box plot showing the levels of ΔmtDNA or +mtDNA (N>3 biological repeats) determined in individual animals of the parental strains, +/1kbΔmtDNA (A, n=14) or +/4kbΔmtDNA (B, n=18). In the boxplot representation, center line, median; box limits, upper and lower quartiles; whiskers, minimum and maximum; points, data. Data were analyzed using the Wilcoxon Mann-Whitney rank sum test ($P<0.001$).
(E) The percent of hatched embryos of N2 (WT) (N=13, n=869), +/1kbΔmtDNA (N=3, n=136) and +/4kbΔmtDNA (N=3, n=133). Data are means ± 1 standard error of the mean (1SE). Data were analyzed using one-way ANOVA followed by a Tukey's *post-hoc* test.
(F) The percent of gravid adults of N2 (WT) (N=5, n=114), +/1kbΔmtDNA (N=3, n=128) and +/4kbΔmtDNA (N=3, n=134) animals at the indicated times after egg laying. Data were analyzed using Cox proportional-hazards regression (Additional file 1: Table S2). Differences in developmental timing were not significant.
(G) Confocal images of mitochondria in body-wall muscle cells. Age synchronized adults of the heteroplasmy strains +/1kbΔmtDNA and +/4kbΔmtDNA stained with MitoTracker Deep Red (24 hours) were washed, fixed, and imaged.
(H-I) Box plot showing the percent of either 1kbΔmtDNA (H) or 4kbΔmtDNA (I) (N>3 biological repeats) determined in individual animals of the heteroplasmic *fzo-1(wt)* cross progeny strains +/1kbΔmtDNA (H, G1wt-G4wt n=17, 15, 16, 16, respectively) and +/4kbΔmtDNA (I, G1wt-G4wt n=20, 20, 20, 20, respectively). In the boxplot representation, center line, median; box limits, upper and lower quartiles; whiskers, minimum and maximum; points, data. Data were analyzed using Fractional regression (Additional file 1: Table S3). Differences in ΔmtDNA levels were not significant.
Individual data values are presented in Additional file 2.

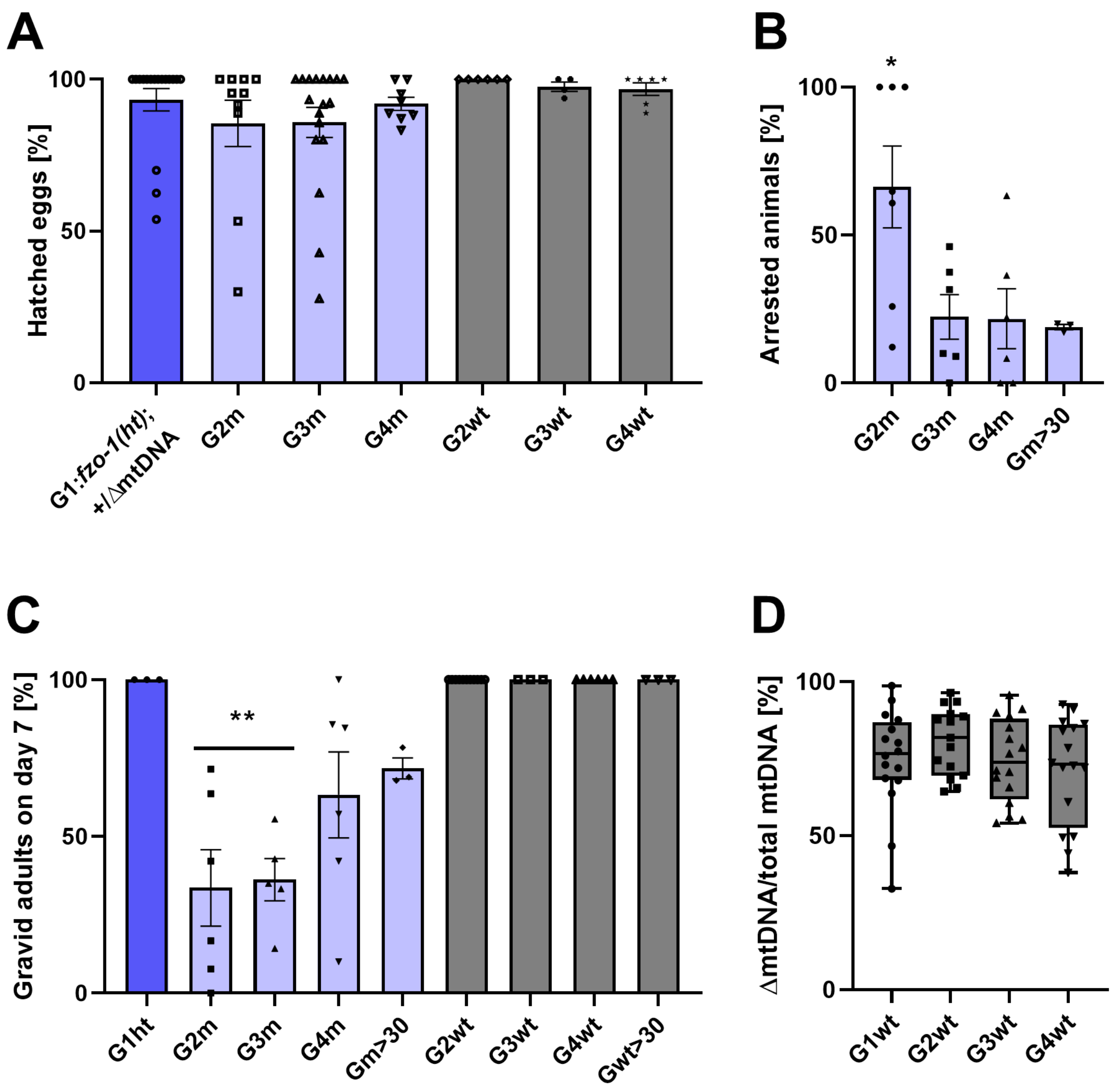

Figure S4: Characterization of ΔmtDNA;*fzo-1(wt);pdr-1(mut)* animals.

(A) The percent of hatched eggs of heteroplasmic *fzo-1(mut);pdr-1(mut);*+/ΔmtDNA or *fzo-1(wt);pdr-1(mut);*+/ΔmtDNA cross progeny monitored across generations (G1ht N=17, n=363; G2m N=9, n=139; G3m N=15, n=284; G4m N=5, n=206; G2wt N=6, n=339; G3wt N=4, n=171; and G4wt N=6, n=235). Data are means ± 1 standard error of the mean (1SE). Data were analyzed using one-way ANOVA followed by a Tukey's *post-hoc* test. Differences in percent of hatching were not significant.

(B) The percent of developmentally arrested animals of *fzo-1(mut);pdr-1(mut);*+/ΔmtDNA mutant cross progeny across generations (G2m N=7, n=152; G3m N=6, n=76; and G4m N=6, n=83) and the stable line (>30 generations, Gm>30 N=3, n=116). Data are means ± 1 standard error of the mean (1SE). Data were analyzed using one-way ANOVA followed by a Tukey's *post-hoc* test. (*) denotes $P<0.05$ by comparison with G4m animals.

(C) The percent of gravid adults seven days after egg laying of heteroplasmic *fzo-1(mut);pdr-1(mut);*+/ΔmtDNA or *fzo-1(wt);pdr-1(mut);*+/ΔmtDNA monitored across

generations (G1ht N=3, n=29; G2m N=6, n=140; G3m N=5, n=83; G4m N=6; n=89; G2wt N=11, n=339; G3wt N=3, n=171; and G4wt N=6, n=235; and stable lines (>30 generations; Gm>30 N=3, n=116; and Gwt>30 N=3, n=78). Data are means ± 1 standard error of the mean (1SE). Data were analyzed using one-way ANOVA followed by a Tukey's *post-hoc* test. (**) denotes P<0.001 by comparison with G1ht animals.

(D) Box plot showing the percent of ΔmtDNA determined (N>3 biological repeats) in individual animals of *fzo-1(wt);pdr-1(mut);*+/ΔmtDNA cross-progeny strains (G1wt-G4wt, n=16, 15, 16 and 16 respectively). In the boxplot representation, center line, median; box limits, upper and lower quartiles; whiskers, minimum and maximum; points, data. Data were analyzed using Fractional regression (Additional file 1: Table S3). Differences in ΔmtDNA levels were not significant.

Individual data values are presented in Additional file 2.
